# Supplementary material for: Trochodendron aralioides, the first chromosome-level draft genome in Trochodendrales and a valuable resource for basal eudicot research
Source: Gigascience. 2019 Nov 18;8(11):giz136. doi: 10.1093/gigascience/giz136 (PMC6859433; doi:10.1093/gigascience/giz136)
Supplement: giz136_GIGA-D-19-00187_Revision_2 [file giz136_giga-d-19-00187_revision_2.pdf]

GigaScience

Trochodendron aralioides, the first chromosome-level draft genome in Trochodendrales and a valuable resource for basal eudicot research

--Manuscript Draft--

**Manuscript Number:**	GIGA-D-19-00187R2
**Full Title:**	Trochodendron aralioides, the first chromosome-level draft genome in Trochodendrales and a valuable resource for basal eudicot research
**Article Type:**	Data Note
**Funding Information:**	Guangxi Province One Hundred Talent program — Assoc Prof Joeri Sergej Strijk<br>Guangxi University — Assoc Prof Joeri Sergej Strijk<br>China Postdoctoral Science Foundation (2015M582481) — Dr Damien Hinsinger<br>China Postdoctoral Science Foundation (2016T90822) — Dr Damien Hinsinger<br>Bagui Scholarship team funding (C33600992001) — Assoc Prof Joeri Sergej Strijk<br>National Natural Science Foundation of China (31470469) — Prof Kunfang Cao
**Abstract:**	Background<br>The wheel tree (Trochodendron aralioides) is one of only two species in the basal eudicot order Trochodendrales. Together with Tetracentron sinense , the family is unique in having secondary xylem without vessel elements, long considered to be a primitive character also found in Amborella and Winteraceae. Recent studies however have shown that Trochodendraceae belong to basal eudicots and demonstrate this represents an evolutionary reversal for the group. Trochodendron aralioides is widespread in cultivation and popular for use in gardens and parks.<br>Findings<br>We assembled the T. aralioides genome using a total of 679.56 Gb of clean reads that were generated using both PacBio and Illumina short-reads in combination with 10XGenomics and Hi-C data. Nineteen scaffolds corresponding to 19 chromosomes were assembled to a final size of 1.614 Gb with a scaffold N50 of 73.37 Mb in addition to 1,534 contigs. Repeat sequences accounted for 64.226% of the genome, and 35,328 protein-coding genes with an average of 5.09 exons per gene were annotated using de novo , RNA-seq, and homology-based approaches. According to a phylogenetic analysis of protein-coding genes, T. aralioides diverged in a basal position relatively to core eudicots, approximately 121.8-125.8 million years ago.<br>Conclusions<br>Trochodendron aralioides is the first chromosome-scale genome assembled in the order Trochodendrales. It represents the largest genome assembled to date in the basal eudicot grade, as well as the closest order relative to the core-eudicots, as the position of Buxales remains unresolved.<br>This genome will support further studies of wood morphology and floral evolution, and will be an essential resource for understanding rapid changes that took place at the base of the Eudicot tree. Finally, it can serve as a valuable source to aid both the acceleration of genome-assisted improvement for cultivation and conservation efforts of the wheel tree.
**Corresponding Author:**	Joeri Sergej Strijk, Ph.D.<br>Guangxi University<br>Nanning, Guangxi CHINA
**Corresponding Author Secondary Information:**	
**Corresponding Author's Institution:**	Guangxi University
**Corresponding Author's Secondary Institution:**	

Powered by Editorial Manager® and ProduXion Manager® from Aries Systems Corporation

**First Author:**	Joeri Sergej Strijk, Ph.D.
**First Author Secondary Information:**	
**Order of Authors:**	Joeri Sergej Strijk, Ph.D.
	Damien Hinsinger, Ph.D.
	Feng-Ping Zhang
	Kunfang Cao, Ph.D.
**Order of Authors Secondary Information:**	

Response to Reviewers:

Joeri Strijk, Damien Hinsinger, Feng-Ping Zhang, KunFang Cao
18th September 2019
Biodiversity Genomics Team
Plant Ecophysiology & Evolution Group
Guangxi Key Laboratory of Forest Ecology and Conservation
College of Forestry, Guangxi University, Nanning, Guangxi 530005 PR China

Dear Editor,

Please find attached our reply to the review of our manuscript entitled "Trochodendron aralioides, the first chromosome-level draft genome in Trochodendrales and a valuable resource for basal eudicot research" by Joeri S. Strijk, Damien D. Hinsinger, Feng-Ping Zhang and KunFang Cao. We have carefully considered the reviewer's opinion and provide here a detailed point-by-point treatment of the remaining points. Please find our responses to the specific comments below, in italics.

The reviewer has maintained some of the previously raised remarks, and we have discussed these (together with our sequence facility personal). We have also looked into common-use practices on both of these, to aid in further clarification. Below we provide additional explanation.

Reviewer reports:
Reviewer #1: The authors generally answered my questions together with changes in the text so I think this paper should be accepted for publication. However, I still have to clarify some issues:

1. An E-value cutoff = 1e-5 for protein search means a piece (HSP) of shorter than 20 bp with low identity would pass. And I don't believe this did not change their results. There is no standard for the e-value cutoff as the authors argued and even if there were, I believe it would not be 1e-5. I can make a much longer list of literatures using more stringent and serious cutoffs from different statistics. I am sorry to say that the so called "standard" used in the studies provided by authors is not so serious.

As an example for a randomly picked sequence blastp against NCBI:
Range 1: 145 to 168GenPeptGraphicsNext MatchPrevious Match
Alignment statistics for match #1

```
Score   Expect  Identities      Positives       Gaps
51.1 bits(113)  1e-05   16/24(67%)      17/24(70%)      0/24(0%)
Query  1    CGNETMKILLGAVEVLWAQQEQEW  24
            CGNETM IL GA E LW  +EQ W
Sbjct  145  CGNETMIILAGALEALWSAHEQNW  168
```

Reply : We disagree with the reviewer for several reasons :
Homology assessment is only one of the methods we used for gene annotations. As detailed in the manuscript, we also used ab initio and transcriptomes, and the results of these 3 approaches were combined and filtered. Thus any misidentified homology can be expected to be corrected during the combination and filtering steps.
The example the reviewer gave cannot be compared with our results, as we blasted nucleotide sequences, not a protein sequence as exemplified, and that even the corresponding nt sequence would have been dropped out during the filtering step "we filtered out low quality gene models, defined as follows: (1) coding region lengths ≤150 bp" (20 amino-acids=90 nucleotides).

Powered by Editorial Manager® and ProduXion Manager® from Aries Systems Corporation

	We agree that some other studies have in fact used a more stringent cutoff value, especially when using closely related species. However, when blasting against distant taxa, as in our manuscript (and other genomes-based studies where no closely related species is available), using a cutoff value of 1E-5 is more common, to take into account the bigger expected genetic distances. As already outlined previously, many studies (several of which published in GigaScience or other high impact journals) relied on this cutoff value.<br><br>2. I am not sure if adding "de novo identified TEs" improved the estimation or just greatly overestimated the TE% due to e.g. very loose cutoff choice by the authors. It almost triple the size. Simply increase the TE family size cannot be called as "improved"<br><br>Reply : The bigger number of de novo identified TEs is due to the different approaches used, as highlighted in both the manuscript and the table legend. To summarize, the TE-proteins were identified using one pipeline that identified ONLY the transposable elements that contain the searched protein domain. On the opposite, the de novo approach used 3 different softwares, based on different methods and algorithms to identify also the non protein TEs. In addition, our values are in the same range than other publications in plants (when different methods are distinguished, e.g. in Ceiba bombax - Gao et al. GigaScience, 7(5), 2018, https://doi.org/10.1093/gigascience/giy051).<br><br>Thank you in advance for considering our manuscript. Please do not hesitate to contact us should you require any additional information. We look forward to hearing from you soon.<br><br>Yours sincerely,<br><br>The authors
Additional Information:	
**Question**	**Response**
Are you submitting this manuscript to a special series or article collection?	No
**Experimental design and statistics**<br><br>Full details of the experimental design and statistical methods used should be given in the Methods section, as detailed in our Minimum Standards Reporting Checklist. Information essential to interpreting the data presented should be made available in the figure legends.<br><br>Have you included all the information requested in your manuscript?	Yes
**Resources**<br><br>A description of all resources used, including antibodies, cell lines, animals and software tools, with enough	Yes

information to allow them to be uniquely identified, should be included in the Methods section. Authors are strongly encouraged to cite Research Resource Identifiers (RRIDs) for antibodies, model organisms and tools, where possible.<br><br>Have you included the information requested as detailed in our Minimum Standards Reporting Checklist?	
**Availability of data and materials**<br><br>All datasets and code on which the conclusions of the paper rely must be either included in your submission or deposited in publicly available repositories (where available and ethically appropriate), referencing such data using a unique identifier in the references and in the "Availability of Data and Materials" section of your manuscript.<br><br>Have you have met the above requirement as detailed in our Minimum Standards Reporting Checklist?	Yes

Powered by Editorial Manager® and ProduXion Manager® from Aries Systems Corporation

Manuscript

Click here to access/download;Manuscript;Strijk_et_al_2019_GigaScience-

Click here to view linked References

Trochodendron aralioides, the first chromosome-level draft genome in Trochodendrales and a valuable resource for basal eudicot research

Joeri S. Strijk[1,2,3†*], Damien D. Hinsinger[2,3†], Fengping Zhang[4], Kunfang Cao[1]

[1] State Key Laboratory for Conservation and Utilization of Subtropical Agro-bioresources, College of Forestry, Guangxi University, Nanning, Guangxi 530005, China

[2] Biodiversity Genomics Team, Plant Ecophysiology & Evolution Group, Guangxi Key Laboratory of Forest Ecology and Conservation, College of Forestry, Daxuedonglu 100, Nanning, Guangxi, 530005, China

[3] Alliance for Conservation Tree Genomics, Pha Tad Ke Botanical Garden, PO Box 959, 06000 Luang Prabang, Laos

[4] Evolutionary Ecology of Plant Reproductive Systems Group, Kunming Institute of Botany, Kunming, China

†Contributed equally to this work.

*Corresponding author: jsstrijk@hotmail.com

ORCIDs:

Joeri S. Strijk, 0000-0003-1109-7015

Damien D. Hinsinger, 0000-0001-7459-7610

Abstract

Background

The wheel tree (*Trochodendron aralioides*) is one of only two species in the basal eudicot order Trochodendrales. Together with *Tetracentron sinense,* the family is unique in having secondary xylem without vessel elements, long considered to be a primitive character also found in *Amborella* and Winteraceae. Recent studies however have shown that Trochodendraceae belong to basal eudicots and demonstrate this represents an evolutionary reversal for the group. *Trochodendron aralioides* is widespread in cultivation and popular for use in gardens and parks.

Findings

We assembled the *T. aralioides* genome using a total of 679.56 Gb of clean reads that were generated using both PacBio and Illumina short-reads in combination with 10XGenomics and Hi-C data. Nineteen scaffolds corresponding to 19 chromosomes were assembled to a final size of 1.614 Gb with a scaffold N50 of 73.37 Mb in addition to 1,534 contigs. Repeat sequences accounted for 64.226% of the genome, and 35,328 protein-coding genes with an average of 5.09 exons per gene were annotated using *de novo*, RNA-seq, and homology-based approaches. According to a phylogenetic analysis of protein-coding genes, *T. aralioides* diverged in a basal position relative to core eudicots, approximately 121.8-125.8 million years ago.

Conclusions

Trochodendron aralioides is the first chromosome-scale genome assembled in the order Trochodendrales. It represents the largest genome assembled to date in the basal eudicot grade, as well as the closest order relative to the core-eudicots, as the position of Buxales remains unresolved.

This genome will support further studies of wood morphology and floral evolution, and will be an essential resource for understanding rapid changes that took place at the base of

the Eudicot tree. Finally, it can serve as a valuable source to aid both the acceleration of genome-assisted improvement for cultivation and conservation efforts of the wheel tree.

Keywords: Trochodendron aralioides; chromosome-level genome assembly; Hi-C assembly; basal eudicot

Data description

Introduction of T. aralioides

The Trochodendraceae family (order Trochodendrales) includes only two species (*Trochodendron aralioides* and *Tetracentron sinense*), both of whom are commercially used and widely cultivated. *Trochodendron aralioides* (or wheel tree) is a native species of the forests of Japan (Honshu – southwards from Yamagata Prefecture, Shikoku, Kyushu, Ryukyu Islands) and Taiwan. Although its hardiness extends to lower temperatures, it is generally restricted to lower temperate montane mixed forests between 600-1700m in Japan. In Taiwan, the range is more extensive, occurring in broad-leaved evergreen forest (2000-3000m) in the central mountain ranges and in northern Taiwan between 500-1250m forming monotypic stands [1] . Over the past century, it has been repeatedly reported from Korea [2–10] although these occurrences are not confirmed in online repositories (e.g. http://plantsoftheworldonline.org/). Properties of *Trochodendron* (e.g. mild-warm temperate range, restricted elevational intervals and natural occurrence in small discontinuous populations) make it difficult to predict the sensitivity of *T. aralioides* to the effects of projected changes in climate [11] . The fossil record shows both species diversity and distribution of the family were much more extensive and continuous during the Eocene (50-52 Ma) to Miocene [12,13] . Unique for basal eudicots, Trochodendraceae have secondary xylem without vessel elements, a property only found in *Amborella* and

Winteraceae [14] . This raises interesting questions on the biological conditions or triggers giving rise to such anatomical reversals, and the evolutionary and ecological consequences inherent in them.

Here, we constructed a high-quality chromosome-level reference genome assembly for *T. aralioides* (NCBI: txid4407) using long reads from the PacBio DNA sequencing platform and a genome assembly strategy taking advantage of the Canu assembler [15] . This assembly of *T. aralioides* genome is the first chromosome-level reference genome constructed for the Trochodendrales order, and the closest relative to core eudicots sequenced to date. The completeness and continuity of the genome will provide high quality genomic resources for studies on floral evolution and the rapid divergence of eudicots.

Genomic DNA extraction, Illumina sequencing and genome size estimation

High-quality genomic DNA was extracted from freshly frozen leaf tissue of *T. aralioides* (Figure 1) using the Plant Genomic DNA Kit (Tiangen Biotech Co., Ltd), following the manufacturer's instructions. After purification, a short-insert library (300~350 bp) was constructed and sequenced on the Illumina NovaSeq platform (Illumina Inc., San Diego, CA), according to manufacturer guidelines. A total of ~124.6 Gb of raw reads were generated.

Sequencing adapters were then removed from the raw reads, and reads from non-nuclear origins (e.g. chloroplast, mitochondrial, bacterial and viral sequences) screened by aligning them to the nr database (NCBI, http://www.ncbi.nlm.nih.gov) using megablast v2.2.26 with the parameters ‘ -v 1 -b 1 -e 1e-5 -m 8 -a 13 ’; The in-house script *duplication_rm.v2* was used to remove the duplicated read pairs; low-quality reads were filtered as follows:

1) reads with ≥10% unidentified nucleotides (N) were removed;

2) reads with adapters were removed;

3) reads with >20% bases having Phred quality <5 were removed;

After the removal of low-quality and duplicated reads, ~124.3 Gb of clean data (Supplementary Table S1) were used for the genome size estimation.

The k-mer peak occurred at a depth of 51 (Figure 2), and we calculated the genome size of *T. aralioides* to be 1.758 Gb, with an estimated heterozygosity of 0.86% and a repeat content of 69.31%. This estimate is slightly smaller than previously reported size, based on cytometry estimate (1.868 Gb) [16] . The GC content was 39.58% (Supplementary Figure S1). A first genome assembly, using the Illumina data and the assembly program SOAPdenovo (SOAPdenovo, RRID:SCR_010752) [17], was approximately 1.324 Gb total length, with a contig N50 of 740 bp and a scaffold N50 of 1.079 kb . This first attempt to assemble the wheel tree genome was of low-quality, likely due to its high genomic repeat content and high heterozygosity level.

PacBio sequencing

High molecular weight Genomic DNA was sheared using a g-TUBE device (Covaris, Brighton, UK) with 20kb settings. Sheared DNA was purified and concentrated with AmpureXP beads (Agencourt, Bioscience Corp., Beverly, MA) and then used for Single-Molecule Real Time (SMRT) bell sequencing library preparation according to manufacturer's protocol (Pacific Biosciences; 20-kb template preparation using BluePippin size selection). Size selected and isolated SMRT bell fractions were purified using AmpureXP beads (Agencourt, Bioscience Corp., Beverly, MA) and these purified SMRT bells were finally used for primer-and polymerase (P6) binding according to manufacturer's protocol (Pacific Biosciences). DNA-Polymerase complexes were used for MagBead binding and loaded at 0.1nM on-plate concentration in 35 SMRT cells. Single-molecule sequencing was performed on a PacBio Sequel platform, yielding a total of 177.80 Gb filtered polymerase read bases (Supplementary Table S1).

10X Genomics sequencing

Libraries were built using a Chromium automated microfluidic system (10X Genomics, San Francisco, CA) that allows the combination of the functionalized gel beads and high molecular weight DNA (HMW gDNA) with oil to form a 'Gel bead in emulsion (GEM)'. Each GEM contains ~10 molecules of HMW gDNA and primers with unique barcodes and P5 sequencing adapters. After PCR amplification, P7 sequencing adapters are added for Illumina sequencing. Data were processed as follows: Firstly, 16 bp barcode sequences and the 7bp random sequences are trimmed from the reads, as well as low quality pairs. We generated a total of 186.95 Gb raw data, and 183.52 Gb clean reads (Supplementary Table S1).

Hi-C sequencing data

To build a Hi-C library [18] , nuclear HMW gDNA from *Trochodendron aralioides* leaves was cross-linked, then cut with the DPNII GATC restriction enzyme, leaving pairs of distally located but physically interacted DNA molecules attached to one another. The sticky ends of these digested fragments were biotinylated and then ligated to each other to form chimeric circles. Biotinylated circles, that are chimeras of the physically associated DNA molecules from the original cross-linking, were enriched, sheared and sequenced on an Illumina platform as described above. After adapter removal and filter of low quality reads, a total of 193.90 Gb clean Hi-C reads. Sequencing quality assessment is shown in Supplementary Table S2.

***De novo* Genome assembly**

Short PacBio reads (<5kb) were first used to correct the PacBio long-reads using the 'daligner' option in FALCON (Falcon, RRID:SCR_016089) [19] , and to generate a

consensus sequence. Following this error correction step, reads overlap was used to construct a directed string graph following Myers' algorithm [20]. Contigs were then constructed by finding the paths from the string graph. Error correction of the preceding assembly was performed using the consensus–calling algorithm Quiver (PacBio Inc., Menlo Park, CA, USA) [21]. Reads were assembled and error-corrected with FALCON and Quiver to generate 4226 contigs with a contig N50 length of 702 Kb and total length of 1.607 Gb.

FragScaff [22] was used for 10X Genomics scaffolding, as follows:

1) Linked reads generated using the 10X Genomics library were aligned with BOWTIE v2 (Bowtie, RRID:SCR_005476) [23] against the consensus sequence of the PacBio assembly, to obtain Super-Scaffolds; 2) With increasing distance to consensus sequence, the number of linked reads supporting scaffolds connection will decrease. Consensus sequences without linked read supports were then filtered and only the consensus sequence supported by linked reads was used for the subsequent assembly. FragScaff scaffolding resulted in 1469 scaffolds, with a scaffold N50 length of 3.38 Mb.

To assess the completeness of the assembled *Trochodendron aralioides* genome, we performed a BUSCO analysis by searching against the plant universal bench marking single-copy orthologs (BUSCO, RRID:SCR_015008, version 3.0) [24] . Overall, 91.4% and 2.8% of the 1440 expected genes were identified in the assembled genome as complete and partial, respectively (Supplementary Table S3). Overall, 94.2% (1,356) genes were found in our assembly. We also assessed the completeness of conserved genes in the *T. aralioides* genome by CEGMA (Core Eukaryotic Genes Mapping Approach) (CEGMA, RRID:SCR_015055) [25] . According to CEGMA, 232 conserved genes in *T. aralioides* were identified which have 93.55% completeness compared to the sets of CEGMA (Supplementary Table S3).

The Hi-C clean data were aligned against the PacBio reads assembly using BWA (BWA,

RRID:SCR_010910) [26] . Only the read pairs with both reads aligned to contigs were considered for scaffolding. For each read pair, its physical coverage was defined as the total bp number spanned by the sequence of reads and the gap between the two reads when mapping to contigs. Per-base physical coverage for each base in the contig was calculated as the number of read pairs' physical coverage it contributes too. Misassembly can be detected by the sudden drop in per-base physical coverage in a contig.

Following the physical coverage of the resulting alignment, any misassembly was split to apply corrections. Using the clustering output, the order and orientation of each contig interaction was assessed on intensity of contig-interaction and the position of the interacting reads. Combining the linkage information and restriction enzyme site, the string graph formulation was used to construct the scaffold graph using LACHESIS [27] , and 1469 scaffolds of our draft genome were clustered to 19 Chromosomes (Supplementary Table S4). The *Trochodendron aralioides* genome information is summarized in Supplementary Tables S5.

Repeat sequences in the wheel-tree genome

Transposable elements in the genome assembly were identified both at the DNA and protein level. RepeatModeler (RepeatModeler, RRID:SCR_015027) [28] , RepeatScout (RepeatScout, RRID:SCR_014653) [29] and LTR_FINDER (LTR_Finder, RRID:SCR_015247) [30] were used to build a *de novo* transposable element library with default parameters. RepeatMasker [31] was used to map the repeats from the *de novo* library against Repbase [32] . Uclust [33] was then used with to the 80-80-80 rule [34] to combined results from above software. At the protein level, RepeatProteinMask in the RepeatMasker package was used to identify TE-related proteins with WU-BLASTX searches against the transposable element protein database. Overlapping transposable elements belonging to the same type of repeats were merged.

Repeat sequences accounted for 64.2% of the *T. aralioides* genome, with 57.2% of the genome identified from the *de novo* repeat library (Table 2). Approximately 53.2% of the *T. aralioides* genome was identified as LTR (most often TEs). Among them, LTRs were the most abundant type of repeat sequences, representing 53.249% of the whole genome. Long interspersed nuclear elements (LINEs) and DNA transposable elements repeats accounted for 0.837% and 2.416% of the whole genome, respectively (Table 2, Supplementary Figure S2).

The tRNA genes were identified by tRNAscan-SE (tRNAscan-SE, RRID:SCR_010835) [35] with the eukaryote set of parameters. The rRNA fragments were predicted by aligning them to *Arabidopsis thaliana* and *Oryza sativa* references rRNA sequences using BlastN (BLASTN, RRID:SCR_001598) (E-value of 1E-10) [36]. The miRNA and snRNA genes were predicted using INFERNAL (Infernal, RRID:SCR_011809) [37] by searching against the Rfam database (release 9.1) (Supplementary Table S6).

Genes annotation

RNA preparation and sequencing

RNA-seq was conducted for four tissue libraries (leaf, stem, bark, and bud) from the same individual as for the genome sequencing and assembly. A total of eight libraries were constructed (Supplementary Table S7). Total RNA was extracted using the RNAprep Pure Plant Kit (TIANGEN, Beijing, PR China) and gDNA contamination was removed with the RNase-Free DNase I (TIANGEN, Beijing, PR China). RNA quality was determined based on the estimation of the ratio of absorbance at 260nm/280nm (OD = 2.0) and the RIN (value = 9.2) by using a Nanodrop ND-1000 spectrophotometer (LabTech, USA) and a 2100 Bioanalyzer (Agilent Technologies, USA), respectively. The cDNA libraries were constructed with the NEBNext Ultra RNA Library Prep Kit for Illumina (New England Biolabs, Ipswich, Massachusetts, MA), following the manufacturer’s recommendations.

Libraries were sequenced on an Illumina HiseqXTen platform (Illumina Inc., San Diego, CA), generating 150-bp paired-end reads. 26.7 Gb of clean RNA-seq sequences were produced, with at least 93.69% of the base with quality >Q20 (Supplementary Table S7). RNA clean reads were both assembled into 312246 sequences using Trinity (Trinity, RRID:SCR_013048) [38] and then annotated, and mapped against the genomic sequence using tophat v2.0.8 (TopHat, RRID:SCR_013035) [39] , then cufflinks v2.1.1 (Cufflinks, RRID:SCR_014597) [40] was used to assemble transcripts into gene models. Finally, all annotation results were combined with EVidenceModeler (EVM) (EVidenceModeler, RRID:SCR_014659) [41] to obtain the final non-redundant gene set.

Annotation

Gene annotation was performed using three approaches:

- A homology-based approach, in which the protein sequences from *O. sativa*, *A. coerulea*, *F. excelsior*, *N. nucifera*, *Q. robur* and *V. vinifera* were aligned to the genome by using TblastN (TBLASTN, RRID:SCR_011822) [36] with an E-value cutoff by 1E-5. Blast hits were conjoined with Solar software [42]. For each blast hit, Genewise (GeneWise, RRID:SCR_015054) [43] was used to predict the exact gene structure in the corresponding genomic regions.
- An *ab initio* gene prediction approach, using Augustus v2.5.5 (Augustus, RRID:SCR_008417) [44] , Genescan v1.0 (GENSCAN, RRID:SCR_012902), GlimmerHMM v3.0.1 (GlimmerHMM, RRID:SCR_002654) [45] , Geneid [46] and SNAP (SNAP, RRID:SCR_007936) [47] to predict coding genes on the repeats-masked *T. aralioides* genome.
- A transcriptome-based approach, in which RNA-seq data were mapped to the genome (see above).

All gene models predicted from the above three approaches were combined by EVM into

a non-redundant set of gene structures. Then, we filtered out low quality gene models, defined as follows: (1) coding region lengths ≤150 bp, (2) models supported only by *ab initio* methods and with FPKM<1.

We identified an average of 5.1 exons per gene (mean length of 10.622 KB) in the *T. aralioides* genome (Table 1). The gene number, gene length distribution, CDS length distribution, exon length distribution and intron length distribution were all comparable to those of selected angiosperms species (Supplementary Table S8, Supplementary Figure S3).

Functional annotation was performed by blasting the protein coding genes sequences against SwissProt and TrEMBL [48] using BLASTP (BLASTP, RRID:SCR_001010) (e-value 1E-5) [49]. The annotation information of the best BLAST hit from the databases were transferred to our gene set annotations. Protein domains were annotated by searching the InterPro(v32.0) and Pfam (V27.0) databases using InterProScan v4.8 (InterProScan, RRID:SCR_005829) [50] and Hmmer v3.1 (Hmmer, RRID:SCR_005305) [51] , respectively. Gene Ontology (GO) terms for each gene were obtained from the corresponding InterPro or Pfam entry. The pathways in which the gene might be involved were assigned by blasting them against the KEGG database (release53), with an E-value cutoff of 1E-5. The genes successfully annotated in GO were classified into Biological process (BP), Cellular component (CC), Molecular Function (MF). Ultimately, 95.4% (33,696 genes) of the 35,328 genes were annotated by at least one database (Supplementary Table S9).

Gene family identification and phylogenetic analyses of wheel-tree

Orthologous relationships between genes of *Amaranthus hypochondriacus*, *Amborella trichopoda*, *Annona muricata*, *Aquilegia coerulea*, *Arabidopsis thaliana*, *Helianthus annuus*, *Cinnamomum kanehirae*, *Musa acuminata*, *Nelumbo nucifera*, *Oryza sativa*,

Quercus robur and *Vitis vinifera* were inferred through all-against-all protein sequence similarity searches with OrthoMCL [52] and only the longest predicted transcript per locus was retained (Supplementary Figure S4, S5). This resulted in 31,290 orthologous groups with representatives in each species being identified. Among these gene families, 484 were single-copy orthologs, and used for phylogenomic reconstruction and positive selection analyses. The similarity among these orthologs ranged from 34.8% to 80.3% (mean: 60.64±7.33 SD, distribution shown in Supplementary Figure S6a).

For each gene family, an alignment was produced using Muscle (MUSCLE, RRID:SCR_011812) [53] , and ambiguously aligned positions trimmed using Gblocks (Gblocks, RRID:SCR_015945) [54] with default parameters (-b3 8;-b4 10;-b5 n) . A Maximum Likelihood (ML) tree was inferred using RAxML 7.2.9 (RAxML, RRID:SCR_006086) [55] .

Divergence times between species were calculated using MCMC, as implemented in PAML (PAML, RRID:SCR_014932) [56] .

Nodes calibrations were defined from the TimeTree database (http://www.timetree.org/) as follows:

- the divergence between *Arabidopsis thaliana* and *Quercus robur* (97–109 Ma);
- the divergence between *Helianthus annuus* and *Amaranthus hypochondriacus* (107-116 Ma);
- the divergence between *Arabidopsis thaliana* and *Vitis vinifera* (109-114 Ma);
- the divergence between *Nelumbo nucifera* and *Vitis vinifera* (116–127 Ma) ;
- the divergence between *Musa acuminata* and *Oryza sativa* (90-115 Ma);
- the divergence between *Arabidopsis thaliana* and *Oryza sativa* (140-200 Ma);
- the divergence between *Amborella Trichopoda* and *Oryza sativa* (168–194 Ma).

The basal eudicot grade's most basal representative, namely *A. coerulea*, diverged from

other angiosperms during the lower Cretaceous 130.2 Ma (126.6-136.2 Ma), while *T. aralioides*, the most recently diverged basal eudicot, diverged from the core-eudicots approximately 124 Ma (121.8-125.8 Ma). Finally, the divergence between rosids and asterids, and thus the crown age of core eudicots was reconstructed as 114.0 Ma (111.3-116.2 Ma).

To identify gene families that experienced a significant expansion or contraction during the evolution of the wheel-tree, we used the likelihood model implemented in CAFE (CAFÉ, RRID:SCR_005983) [57] , with default parameters. The phylogenetic tree topology and branch lengths were taken into account to infer the significance of change in gene family size in each branch (Figure 4). The genes families that experienced the most significant expansions were mainly involved in pathogen/stress response [e.g. the cyanoamino-acid metabolism, $p=2.35x10^{-28}$; the plant-pathogen interaction map, $p=2.29x10^{-22}$; the tryptophan metabolism, $p=5.85x10^{-10}$] (Supplementary Table S10).

Positive selection

Positive selection is a major driver of biological adaptation. Using the protein-coding sequences, we calculated the number of synonymous substitutions per site (Ks) and nonsynonymous substitutions per site (Ka) and assessed the deviation from zero of the difference Ka − Ks. A Ka/Ks > 1 represent an evidence of positive selection. The Ka/KS ratio ranged from 0.0061 to 5.7689 (mean: 0.6976 ±0.9173 SD, see Supplementary Figure S6b) with Ks ranging from 0.0030 to 0.6281 (mean: 0.1585 ±0.0749 SD). MUSCLE [53] was used to align the protein and nucleotide sequences, then we used Gblocks [54] with default parameters (-b3 8;-b4 10;-b5 n) to eliminate poorly aligned positions and divergent regions from the alignment. The maximum likelihood-based branch lengths test of PAML package [56] was used for comparisons, and the ratio Ka/Ks was calculated over the entire length of the protein coding gene. Using *T. aralioides* as the foreground branch and

Amaranthus hypochondriacus, Helianthus annuus, Nelumbo nucifera, Aquilegia coerulea as background branches, we identified 238 genes were considered as candidate genes under positive selection (p-value<0.01, FDR < 0.05) using a maximum likelihood-based branch lengths test. The GO terms and KEGG pathways for these genes showed that positive selection was especially detected in cell metabolism (such as vitamins and amino-acid biosynthesis, Supplementary Table S11).

Whole-genome duplication analysis

MCScan [58] was used to identify collinear segments within *Trochodendron* and between the *T. aralioides* and other angiosperm genomes. The sequences of the gene pairs contained in the (inter-) collinear segments of the genome were extracted and the *codeml* tool in the PAML package [56] was used to calculate the value of 4dTv. The distribution of 4dTv can reflect whether genome-wide replication events occur in the evolutionary history of species, the relative time of genome-wide replication events, and the divergent events among species.

The 4dTv values of all paralogous gene pairs in *Trochodendron aralioides* were calculated, as well as those in *Aquilegia coerulea*, *Helianthus annuus* and *Annona muricata* for comparisons. In addition, the 4dTv values were calculated for all ortholog gene pairs between *Trochodendron aralioides* and *A. coerulea, H. annuus* and *A. muricata* to observe species divergence events. The peak of 4dTv distribution in the *T. aralioides* genome was around ~0.1, whereas the peak for interspecific comparisons with *A. coerulea* and *H. annuus* were around ~0.3 and ~0.2, respectively (Supplementary Figure S7). All together, these 4dTv distributions indicate that a whole genome replication event occurred in *T. aralioides* after its divergence from both other basal angiosperms and core-eudicots.

Conclusions

We successfully assembled the genome of *T. aralioides* and report the first chromosome-level genome sequencing, assembly and annotation based on long reads from the third-generation PacBio Sequel sequencing platform for basal eudicotyledons. The final draft genome assembly is approximately 1.614 Gb, which is slightly smaller than both the estimated genome size (1.758 Gb) based on k-mer analysis and on cytometry (1.868 Gb, [16]). With a contig N50 of 691 Kb and a scaffold N50 of 73.37 Mb, the chromosome-level genome assembly of *T. aralioides* is the first high-quality genome in the Trochodendrales order. We also predicted 35,328 protein-coding genes from the generated assembly, and 95.4% (33,696 genes) of all protein-coding genes were annotated. We found that the divergence time between *T. aralioides* and its common ancestor with the core eudicots was approximately 124.2 Ma. The chromosome-level genome assembly together with gene annotation data generated in this work will provide a valuable resource for further research on floral morphology diversity, on the early evolution of eudicotyledons and on the conservation of this iconic tree species.

Availability of supporting data

Raw reads were deposited to EBI (project PRJEB32669), and supporting data and materials are available in the *GigaScience* GigaDB database [59].

Abbreviation

BUSCO: Benchmarking Universal Single-Copy Orthologs; CDS: Coding sequence; GO: Gene ontology; KEGG: Kyoto Encyclopaedia of Genes and Genomes; LINE: Long interspersed nuclear elements; LTR: Long terminal repeats; NGS: Next Generation Sequencing; TE: Transposable elements.

Competing interests

The authors declare that they have no competing interests.

Funding

Genome sequencing, assembly and annotation were conducted by the Novogene Bioinformatics Institute, Beijing, China; mutual contract No. NHT161060. This work was supported through the Bagui Scholarship team funding under Grant No. C33600992001, the Guangxi Province One Hundred Talent program and Guangxi University to JSS, and by funding from the China Postdoctoral Science Foundation under Grant No. 2015M582481 and No. 2016T90822 to DDH, and the National Science Foundation of China under Grant No. 31470469 to KFC.

References

1. Li, HL and Chaw S. Trochodendraceae. Flora of Taiwan. 1996. p.504–5.

2. Ohwi J. Flora of Japan. Washington, DC: Smithsonian Institution; 1965.

3. Hogan S. Trees for all seasons: broadleaved evergreens for temperate climates. Portland, OR: Timber Press; 2008.

4. Mabberley DJ. Mabberley's plant-book: a portable dictionary of plants, their classification and uses. 3rd ed. Cambridge University Press; 2008.

5. Hilliers J, Coombes A. The Hilliers Manual of Trees and Shrubs. Devon: David & Charles; 2002.

6. Phillips R, Rix M. The Botanical Garden: Trees and shrubs. Richmond Hill: Firefly Books; 2002.

7. A.L. J. North American landscape trees. Berkeley: Ten speed press; 1996.

8. Krussman G. Trochodendron. Man Cultiv broad-leaved trees shrubs. Portland, OR: Timber Press; 1985.

9. Bean WJ. Trochodendron. Trees shrubs hardy Br Isles. 8th ed. John Murray; 1980.

10. Rehder A. Manual of cultivated trees and shrubs. New-York: The Macmillan Company; 1940.

11. Larsen T, Brehm G, Navarrete H, Franco P, Gomez H. Range shifts and extinctions driven by climate change in the tropical Andes: synthesis and directions. Clim Chang Biodivers Trop Andes. 2011. p. 348.

12. Manchester SR, Pigg KB, Kvaček Z, DeVore ML, Dillhoff RM. Newly Recognized Diversity in Trochodendraceae from the Eocene of Western North America. Int J Plant Sci. 2018;179:663–76.

13. Manchester SR, Pigg KB, Devore ML. Trochodendraceous fruits and foliage in the Miocene of western North America. Foss Impr. 2018;74:45–54.

14. Cronk QCB, Forest F. The Evolution of Angiosperm Trees: From Palaeobotany to Genomics. Plant Genet Genomics Crop Model. Cham: Springer; 2017. p. 1–17.

15. Koren S, Walenz BP, Berlin K, Miller JR, Bergman NH, Phillippy AM. Canu: Scalable and accurate long-read assembly via adaptive K-mer weighting and repeat separation. Genome Res. 2017;27:722–36.

16. Hanson L, Brown RL, Boyd A, Johnson MAT, Bennett MD. First nuclear DNA C-values for 28 angiosperm genera. Ann Bot. 2003;91:31–8.

17. Luo R, Liu B, Xie Y, Li Z, Huang W, Yuan J, et al. SOAPdenovo2: an empirically improved memory-efficient short-read de novo assembler. Gigascience. 2012;1:18.

18. Lieberman-Aiden E, Van Berkum NL, Williams L, Imakaev M, Ragoczy T, Telling A, et al. Comprehensive mapping of long-range interactions reveals folding principles of the human genome. Science. 2009;326:289–93.

19. Chin C-S, Peluso P, Sedlazeck FJ, Nattestad M, Concepcion GT, Clum A, et al. Phased

diploid genome assembly with single-molecule real-time sequencing. Nat Methods. 2016;13:1050–4. 20. Myers EW. The fragment assembly string graph. Bioinformatics. 2005;21(suppl_2):ii79-6.

21. Chin CS, Alexander DH, Marks P, Klammer AA, Drake J, Heiner C, et al. Non hybrid, finished microbial genome assemblies from long-read SMRT sequencing data. Nat Methods. 2013;10:563-6.

22. Adey A, Kitzman JO, Burton JN, Daza R, Kumar A, Christiansen L, Ronaghi M, Amini S, Gunderson KL, Steemers FJ, Shendure J. In vitro, long-range sequence information for de novo genome assembly via transposase contiguity. Genome Res. 2014 Dec;24(12):2041-9. doi: 10.1101/gr.178319.114.

23. Langmead B, Salzberg SL. Fast gapped-read alignment with Bowtie 2. Nat Methods. 2012;9:357–66.

24. Simão FA, Waterhouse RM, Ioannidis P, Kriventseva E V., Zdobnov EM. BUSCO: Assessing genome assembly and annotation completeness with single-copy orthologs. Bioinformatics. 2015;31:3210–2.

25. Parra G, Bradnam K, Korf I. CEGMA: A pipeline to accurately annotate core genes in eukaryotic genomes. Bioinformatics. 2007;23:1061–7.

26. Li H, Durbin R. Fast and accurate short read alignment with Burrows-Wheeler transform. Bioinformatics. 2009;25:1754–60.

27. Burton JN, Adey A, Patwardhan RP, Qiu R, Kitzman JO, Shendure J. Chromosome-scale scaffolding of de novo genome assemblies based on chromatin interactions. Nat Biotechnol. 2013;31:1119–54.

28. Smit, AFA, Hubley, R. RepeatModeler Open-1.0. 2018 http://www.repeatmasker.org

29. Price AL, Jones NC, Pevzner PA. De novo identification of repeat families in large genomes. Bioinformatics. 2005;21:i351–8.

30. Xu Z, Wang H. LTR-FINDER: An efficient tool for the prediction of full-length LTR retrotransposons. Nucleic Acids Res. 2007;35:W265–8.

31. Tarailo-Graovac M, Chen N. Using RepeatMasker to identify repetitive elements in genomic sequences. Curr Protoc Bioinforma. 2009;25:4–10.

32. Smit A, Hubley R, Green P. RepeatMasker Open-4.0.6 2013-2015. http://www.repeatmasker.org. 2017.

33. Edgar RC. Search and clustering orders of magnitude faster than BLAST. Bioinformatics. 2010;26:2460–1.

34. Wicker T, Sabot F, Hua-Van A, Bennetzen JL, Capy P, Chalhoub B, et al. A unified classification system for eukaryotic transposable elements. Nat Rev Genet. 2007;8:973.

35. Lowe TM, Eddy SR. TRNAscan-SE: A program for improved detection of transfer RNA genes in genomic sequence. Nucleic Acids Res. 1996;25:955–64.

36. Camacho C , Coulouris G, Avagyan V, Ma N, Papadopoulos J, Bealer Ke, t al. 2009, BLAST+: architecture and applications. *BMC Bioinformatics*. 10:421–9.

37. Nawrocki EP, Eddy SR. Infernal 1.1: 100-fold faster RNA homology searches. Bioinformatics. 2013;29:2933–5.

38. Grabher MG, Haas BJ, Yassour M, Levin JZ, Thompson DA, Amit I, et al. Trinity: reconstructing a full-length transcriptome without a genome from RNA-Seq data. Nat Biotechnol. 2013;29:644.

39. Kim D, Pertea G, Trapnell C, Pimentel H, Kelley R, Salzberg SL. TopHat2: accurate alignment of transcriptomes in the presence of insertions, deletions and gene fusions. Genome Biol. 2013;14:R36.

40. Ghosh S, Chan CK. Analysis of RNA-Seq Data Using TopHat and Cufflinks. Methods Mol Biol. 2016;1374:339-61. doi: 10.1007/978-1-4939-3167-5_18.

41. Haas BJ, Salzberg SL, Zhu W, Pertea M, Allen JE, Orvis J, et al. Automated eukaryotic gene structure annotation using EVidenceModeler and the Program to Assemble Spliced Alignments. Genome Biol. 2008;9:1.

42. Yu X, Zheng H, Wang J, et al. Detecting lineage-specific adaptive evolution of brain-expressed genes in human using rhesus macaque as outgroup. Genomics. 2006;88:745–51.

43. Birney E, Clamp M, Durbin R. GeneWise and Genomewise. Genome Res. 2004;14:988–95.

44. Stanke M, Keller O, Gunduz I, Hayes A, Waack S, Morgenstern B. AUGUSTUS: Ab initio prediction of alternative transcripts. Nucleic Acids Res. 2006;32:W309–12.

45. Majoros WH, Pertea M, Salzberg SL. TigrScan and GlimmerHMM: Two open source ab initio eukaryotic gene-finders. Bioinformatics. 2004;20:2878–9.

46. Blanco E, Parra G, Guigó R. Using geneid to identify genes. Curr Protoc Bioinforma. 2007;18:4–7.

47. Johnson AD, Handsaker RE, Pulit SL, Nizzari MM, O'Donnell CJ, De Bakker PIW. SNAP: A web-based tool for identification and annotation of proxy SNPs using HapMap. Bioinformatics. 2008;24:2938–9.

48. UniProt Consortium. UniProt: a hub for protein information. Nucleic Acids Res. 2014;43:D204-8.

49. Gish W, & States DJ. Identification of protein coding regions by database similarity search. Nat Genet. 1993;3:266-6.

50. Quevillon E, Silventoinen V, Pillai S, Harte N, Mulder N, Apweiler R, et al.

InterProScan: Protein domains identifier. Nucleic Acids Res. 2005;33:W116–20.

51. Finn RD, Clements J, Eddy SR. HMMER web server: Interactive sequence similarity searching. Nucleic Acids Res. 2011;39:W29–37.

52. Li L, Stoeckert CJ, Roos DS. OrthoMCL: Identification of ortholog groups for eukaryotic genomes. Genome Res. 2003;13:2178–89.

53. Edgar RC. MUSCLE: multiple sequence alignment with high accuracy and high throughput. Nucleic Acids Res. 2004 Mar 19;32(5):1792-7.

54. Castresana J. Selection of conserved blocks from multiple alignments for their use in phylogenetic analysis. Mol Biol Evol. 2000;17:540–52.

55. Stamatakis A. RAxML version 8: A tool for phylogenetic analysis and post-analysis of large phylogenies. Bioinformatics. 2014;30:1312–3.

56. Yang Z. PAML 4: Phylogenetic analysis by maximum likelihood. Mol Biol Evol. 2007;24:1586–91.

57. De Bie T, Cristianini N, Demuth JP, Hahn MW. CAFE: A computational tool for the study of gene family evolution. Bioinformatics. 2006;22:1269–71.

58. Wang Y, Tang H, Debarry JD, Tan X, Li J, Wang X, et al. MCScanX: A toolkit for detection and evolutionary analysis of gene synteny and collinearity. Nucleic Acids Res. 2012;40:e49–e49.

59. Strijk JS; Hinsinger D; Zhang F; Cao K (2019): Supporting data for "Trochodendron aralioides, the first chromosome-level draft genome in Trochodendrales and a valuable resource for basal eudicot research" GigaScience Database. http://dx.doi.org/10.5524/100657

Tables

Table 1. Summary of *Trochodendron aralioides* genome assembly and annotation.

	**draft scaffolds**	**chromosome-length scaffolds based on Hi-C**
**Genome assembly**		
length of genome (bp)	1623741898	1530107441
Number of contigs	4226	2744
Contigs N50 (bp)	702251	740603
Number of scaffolds	1469	19
Scaffold N50 (bp)	3938440	73365148
Genome coverage (X)	278.34	398.07
Number of contigs (>100kbp)	3062	2744
Total length of contigs (>100kbp)	1567464199	1523319687
Mapping rate of contigs	0.9779	/
**Genome annotation**		
Protein-coding gene number		35,328
Mean transcript length (kb)		10,622.49
Mean exons per gene		5.09
Mean exon length (bp)		232.46

Mean intron length (bp)		2,308.46

Table 2. Detailed classification of repeat sequences identified in *Trochodendron aralioides*. *De novo* + Repbase: annotations predicted *de novo* by RepeatModeler, RepeatScout and LTR_FINDER; TE proteins: transposon elements annotated by RepeatMasker; Combined TEs: merged results from approaches above, with overlap removed. Unknown: repeat sequences RepeatMasker cannot classified.

	de novo **+ Repbase**		**TE proteins**		**Combined TEs**	
**Type**	**Length (bp)**	**% in genome**	**Length (bp)**	**% in genome**	**Length (bp)**	**% in genome**
DNA	38995970	2.416	15755819	0.976	50171660	3.108
LINE	13503128	0.837	39566384	2.451	47644917	2.952
SINE	143207	0.00887	0	0	143207	0.00887
LTR	859515257	53.249	327748739	20.305	908751606	56.23
Unknown	13395729	0.83	0	0	13395729	0.83
**Total**	922704692	57.164	382460417	23.694	1006355712	62.347

Figure legends

Figure 1. *Trochodendron aralioides* description. a) Geographic distribution. light blue:

occurrence according to the Flora of China (at country level), green: occurrence according to GBIF; b) flowers; c) bud; d) general habit; e-f) stem and sprouting bud showing the wheel-like organisation of leaves.

Figure 2. *k*-mer distribution of the *T. aralioides* genome. a) K-mer depth and number frequency distribution; b) K-mer depth and K-mer species number frequency distribution.

Figure 3. Hi-C interaction heat map for *T. aralioides* reference genome showing interactions between the 19 chromosomes.

Figure 4. Phylogenetic tree and number of gene families displaying expansion and contraction among 12 plant species. The pie charts show the expansion (green), contraction (red) and conserved (blue) gene family proportions among all gene families. Estimated divergence time confidence interval are shown at each internal node as teal bars. Calibrated nodes indicated by red dots (see text for details on calibration scheme).

Supplementary Materials

Supplementary Figure S1. GC content analysis of *T. aralioides* genome based on Illumina reads for genome size survey.

Supplementary Figure S2. Divergence distribution of transposable elements in the genome of *T. aralioides*. Units in Kimura substitution level (CpG adjusted).

Supplementary Figure S3. Genes characteristics in *T. aralioides* and other angiosperms. From left to right and top to bottom: lengths of messenger RNA; lengths of exons in coding regions; number of exons per gene; lengths of introns in genes; lengths of coding regions (CDS). Aco: *Aquilegia caeruleus*; Fex: *Fraxinus excelsior*; Nun: *Nelumbo nucifera*; Osa: *Oryza sativa*; Qro: *Quercus robur*; Tar: *Trochodendron aralioides*; Vvi: *Vitis vinifera*.

Supplementary Figure S4. Comparing orthogroups between *T. aralioides* and other angiosperms species. Aco: *Aquilegia caeruleus*; Ahy: *Amaranthus hypochondriacus*; Amu:

Annona muricata; Ath: *Arabidopsis thaliana*; Atr: *Amborella Trichopoda*; Cka: *Cinnamomum micranthum*; Han: *Helianthus annuus*; Mac: *Musa acuminata*; Nnu: *Nelumbo nucifera*; Osa: *Oryza sativa*; Qro: *Quercus robur*; Tar: *Trochodendron aralioides*; Vvi: *Vitis vinifera*.

Supplementary Figure S5. Orthologous gene families across four angiosperms genomes (*Trochodendron aralioides*, *Annona muricata*, *Amaranthus hypochondriacus* and *Aquilegia coerulea*).

Supplementary Figure S6. Characteristics of the 484 orthologs found between *T. aralioides* and selected angiosperms (see text for details). a) Distribution of the similarity among each ortholog; Genes are counted vertically (red bars) for bins of 1% unit (horizontally). b) Distribution of the genes Ka/Ks ratio; Genes are counted vertically (red bars) for bins of 0.1 Ka/Ks unit (horizontally).

Supplementary Figure S7. Distribution of 4dTv values in several species pairs comparisons, highlighting potential Whole Genome Duplications (WGD). Aco: *Aquilegia caeruleus*; Amu: *Annona muricata;* Han: *Helianthus annuus*.

Figure 1 Click here to access/download;Figure;Figure_1-description_Trochodendron.pdf

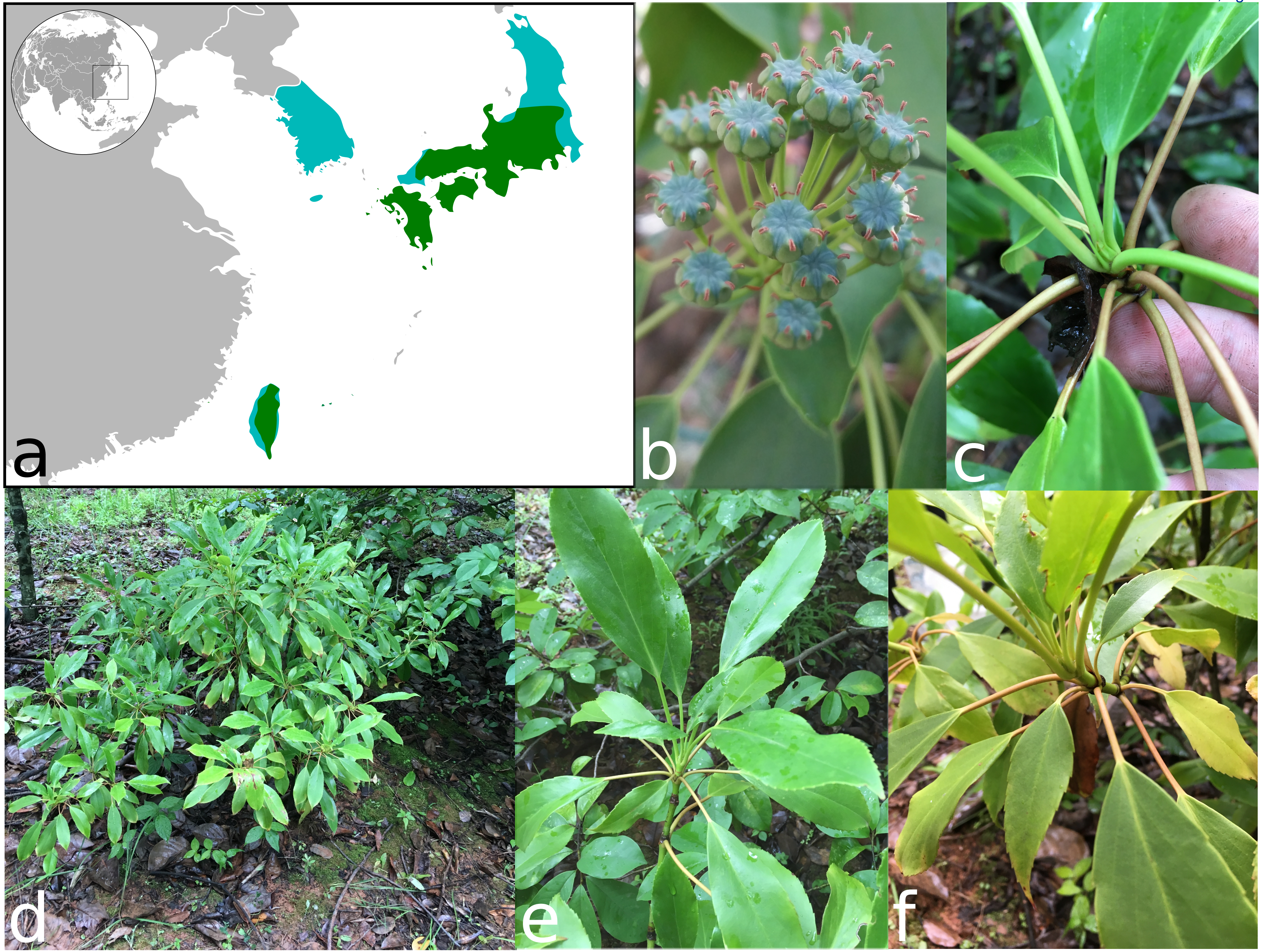

Figure 2

Click here to access/download;Figure;Figure_2-kmers_distribution.pdf

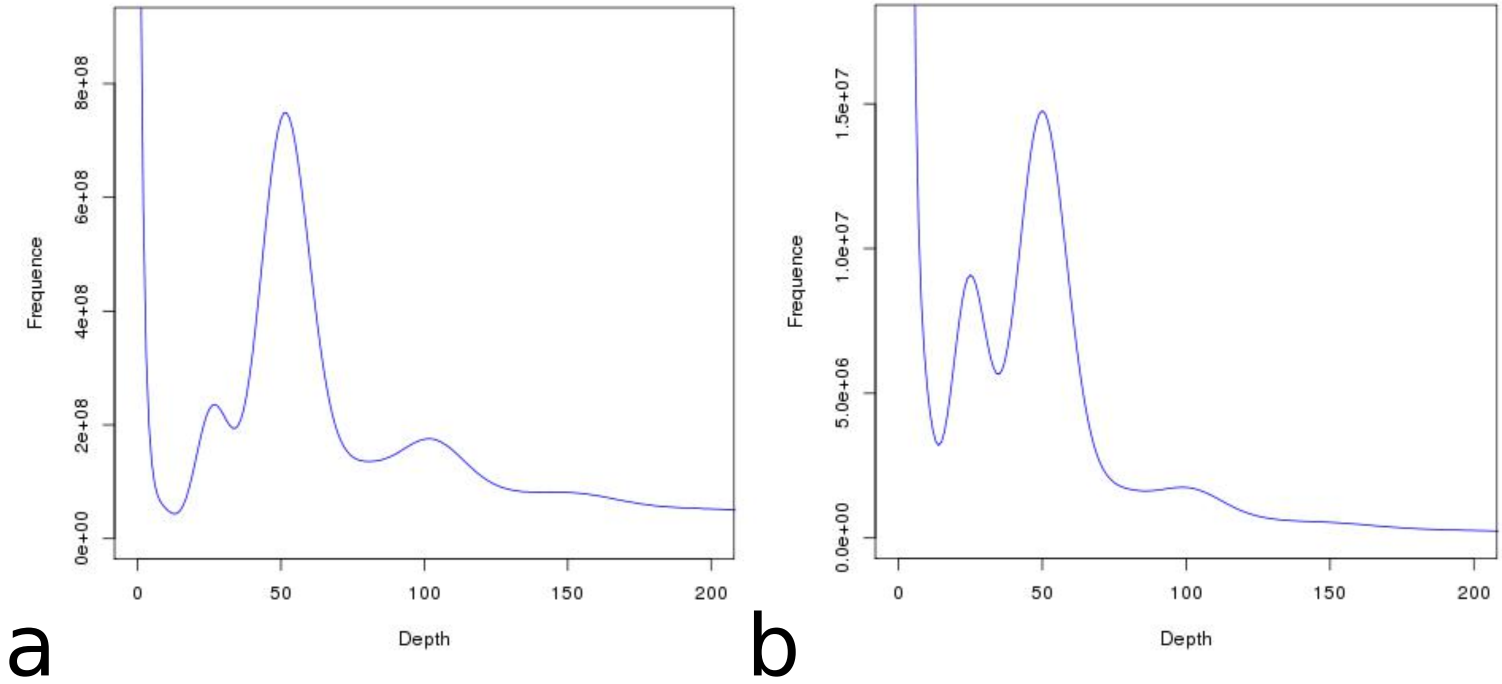

Figure 3

Click here to access/download;Figure;Figure_3-

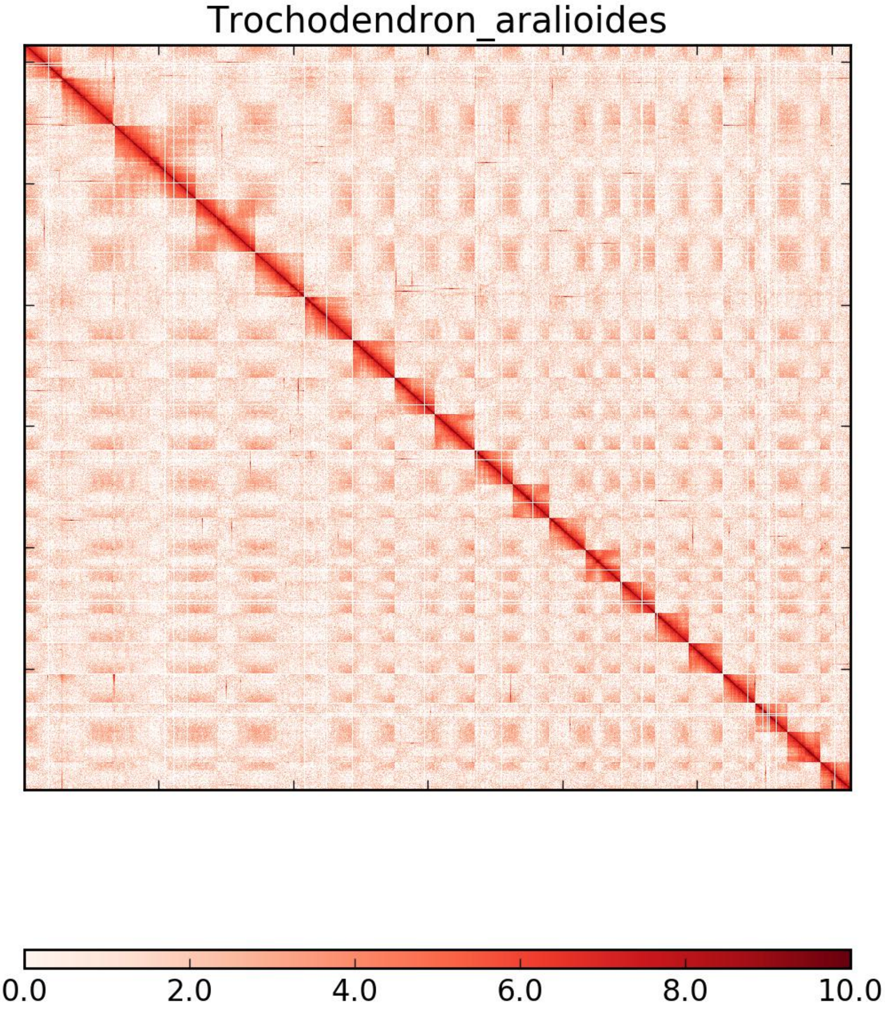

Figure 4

Click here to access/download;Figure;Figure_4-phylogenetic_tree.pdf

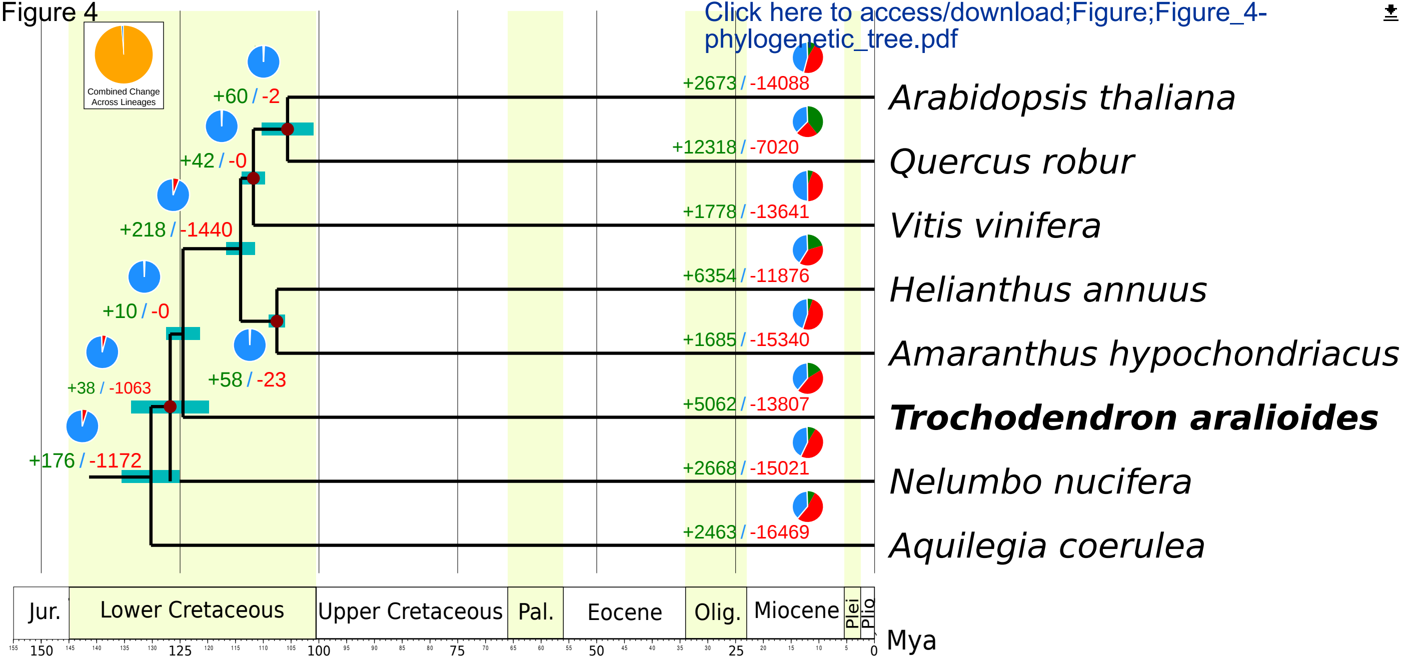

Supplementary Material Figure 1

Click here to access/download
Supplementary Material
Supp_Figure_1-GC_content.pdf

Supplementary Material Figure 2

Click here to access/download
Supplementary Material
Supp_Figure_2-repeat_content.pdf

Supplementary Material Figure 3

Click here to access/download

Supplementary Material

Supp_Figure_3-genes_characteristics.pdf

Supplementary Material Figure 4

Click here to access/download
Supplementary Material
Supp_Figure_4-comparative_orthogroups.pdf

Supplementary Material Figure 5

Click here to access/download
Supplementary Material
Supp_Figure_5-orthologs.pdf

Supplementary Material Figure 6

Click here to access/download
Supplementary Material
Supp_Figure_6-orthologs_features.pdf

Supplementary Material Figure 7

Click here to access/download
Supplementary Material
Supp_Figure_7-4dTv.pdf

Supplementary Material Tables

Click here to access/download
Supplementary Material
Supplementary_tables.docx
