# Supplementary figures and images for: Trochodendron aralioides, the first chromosome-level draft genome in Trochodendrales and a valuable resource for basal eudicot research
Source: Gigascience. 2019 Nov 18;8(11):giz136. doi: 10.1093/gigascience/giz136 (PMC6859433; doi:10.1093/gigascience/giz136)

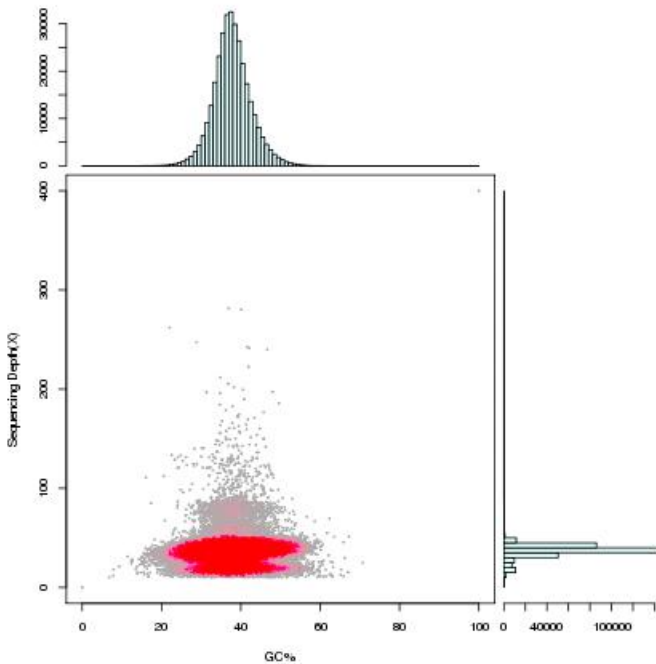

Supplement: giz136_Supplemental_Figures_and_Tables [file giz136_supplemental_figures_and_tables.zip › Supp_Figure_1-GC_content.pdf]

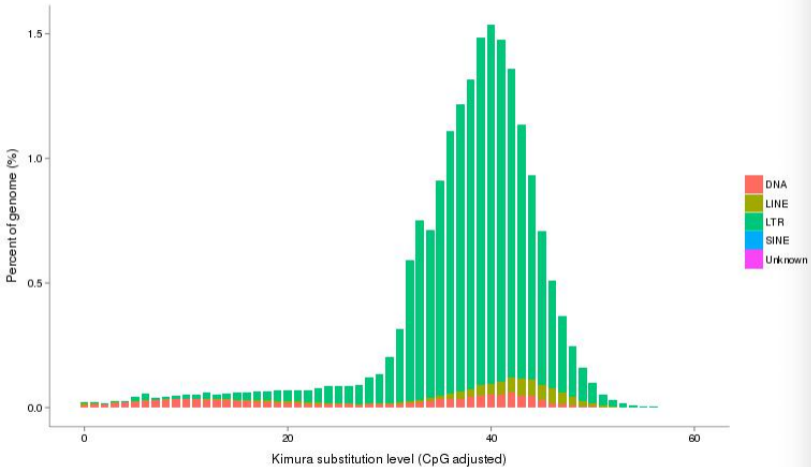

Supplement: giz136_Supplemental_Figures_and_Tables [file giz136_supplemental_figures_and_tables.zip › Supp_Figure_2-repeat_content.pdf]

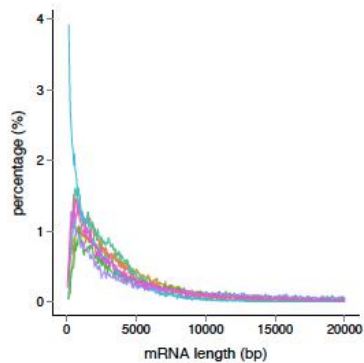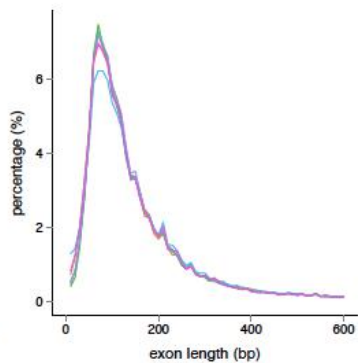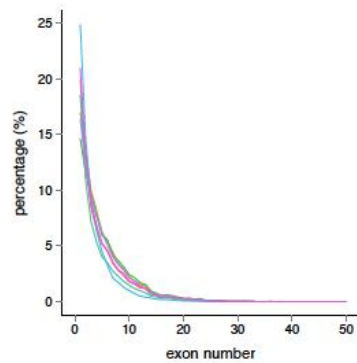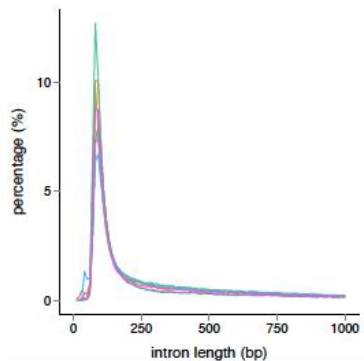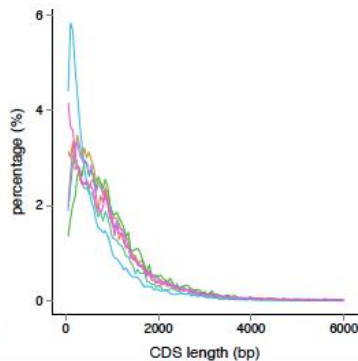

species

Aco  
Fax  
Nun  
Osa  
Qro  
Tar  
Vvi

Supplement: giz136_Supplemental_Figures_and_Tables [file giz136_supplemental_figures_and_tables.zip › Supp_Figure_3-genes_characteristics.pdf]

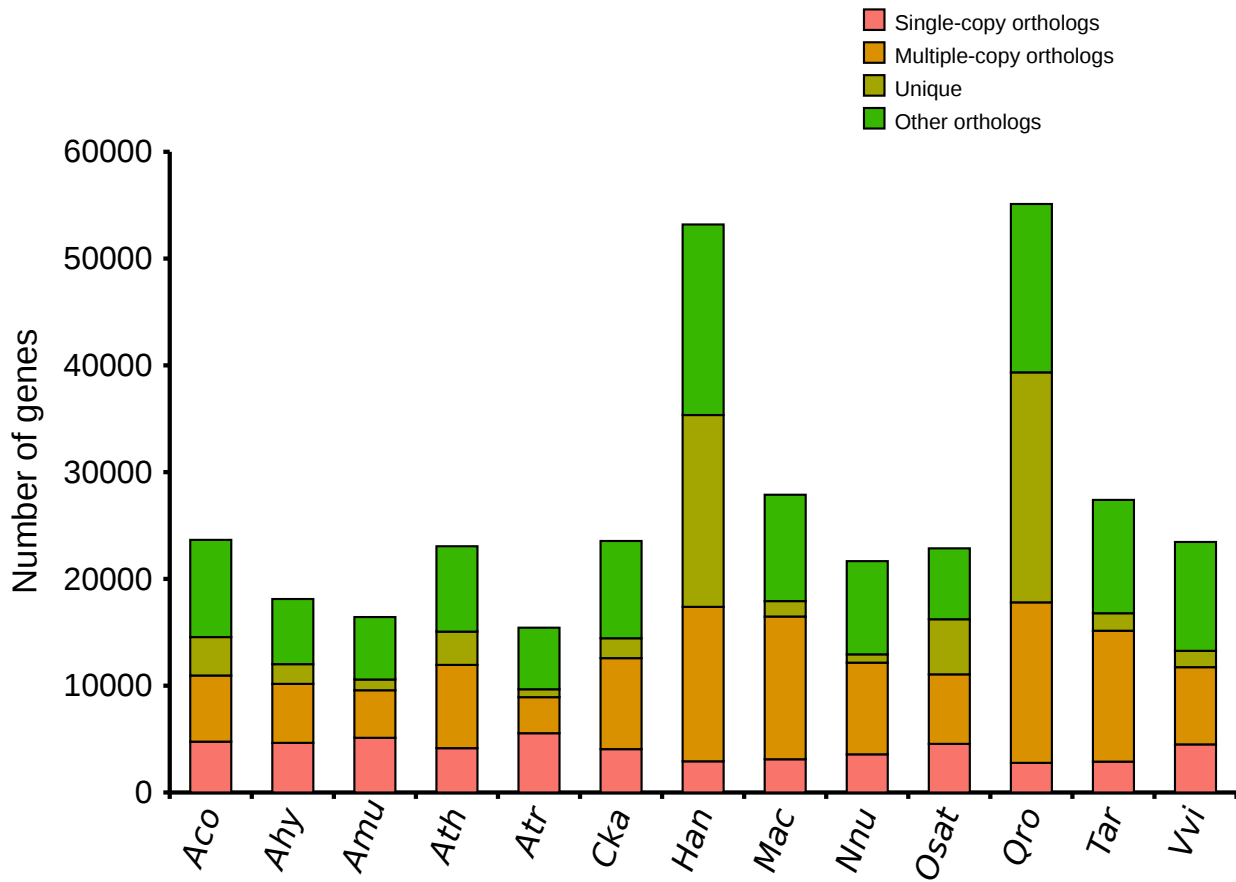

Supplement: giz136_Supplemental_Figures_and_Tables [file giz136_supplemental_figures_and_tables.zip › Supp_Figure_4-comparative_orthogroups.pdf]

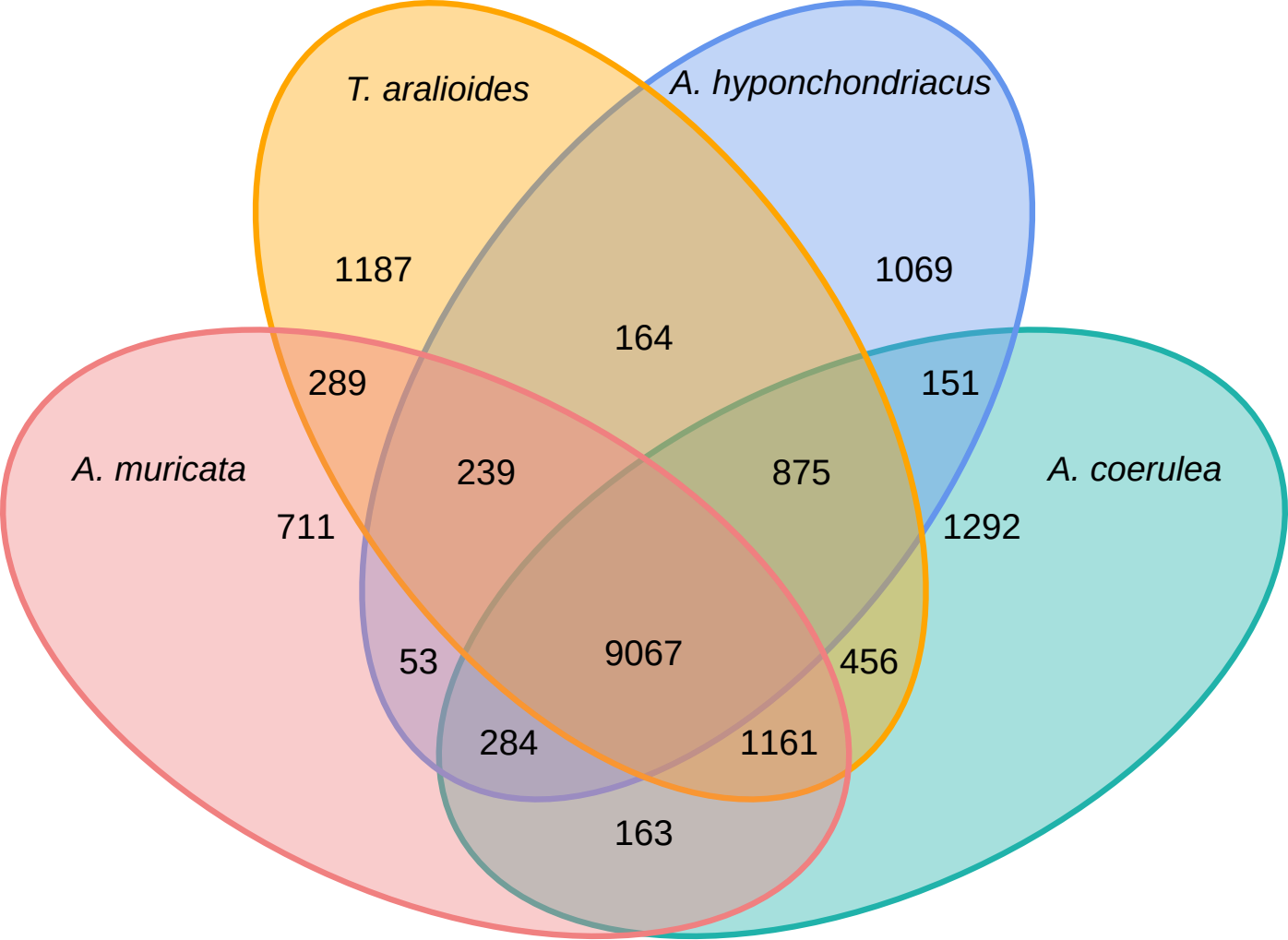

Supplement: giz136_Supplemental_Figures_and_Tables [file giz136_supplemental_figures_and_tables.zip › Supp_Figure_5-orthologs.pdf]

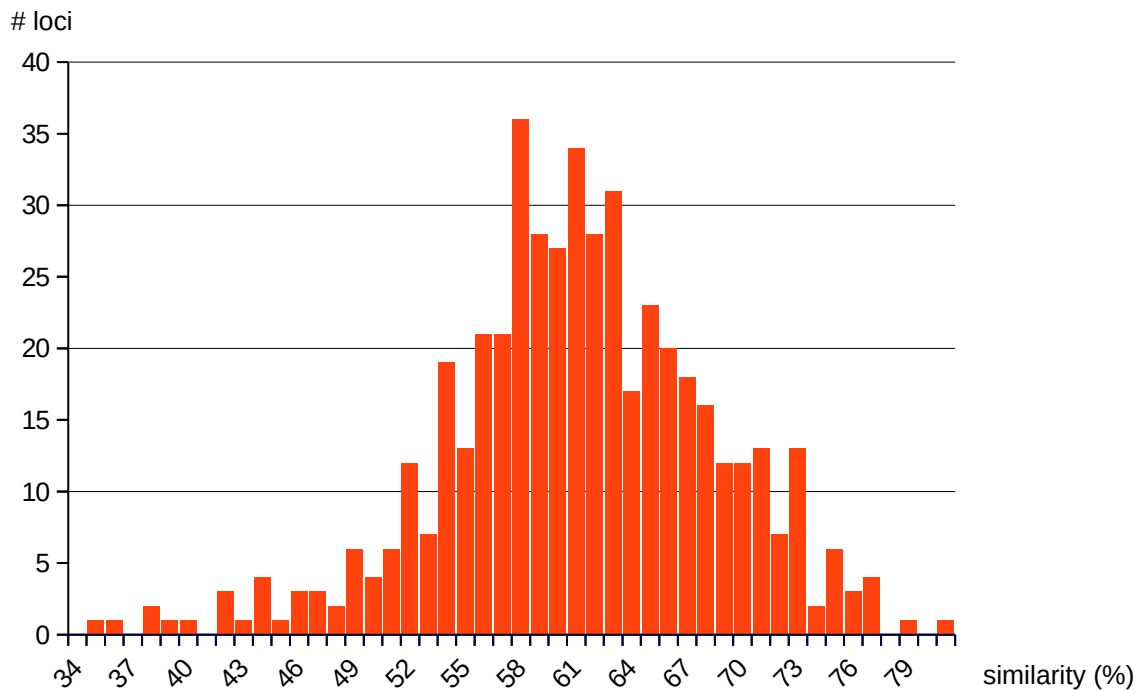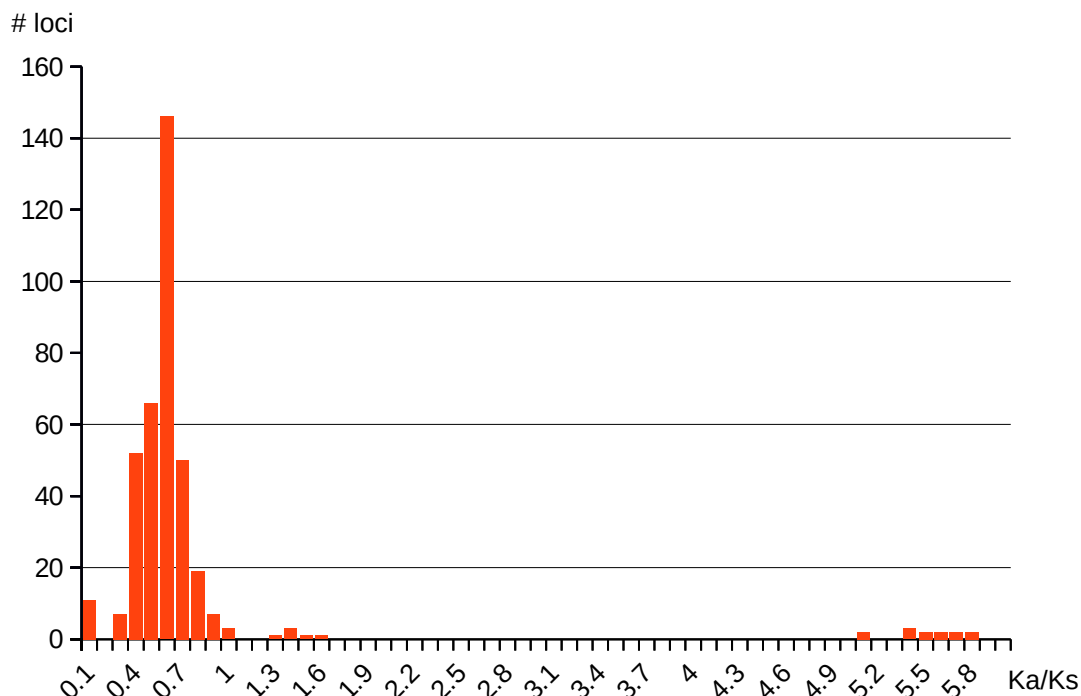

Supplement: giz136_Supplemental_Figures_and_Tables [file giz136_supplemental_figures_and_tables.zip › Supp_Figure_6-orthologs_features.pdf]

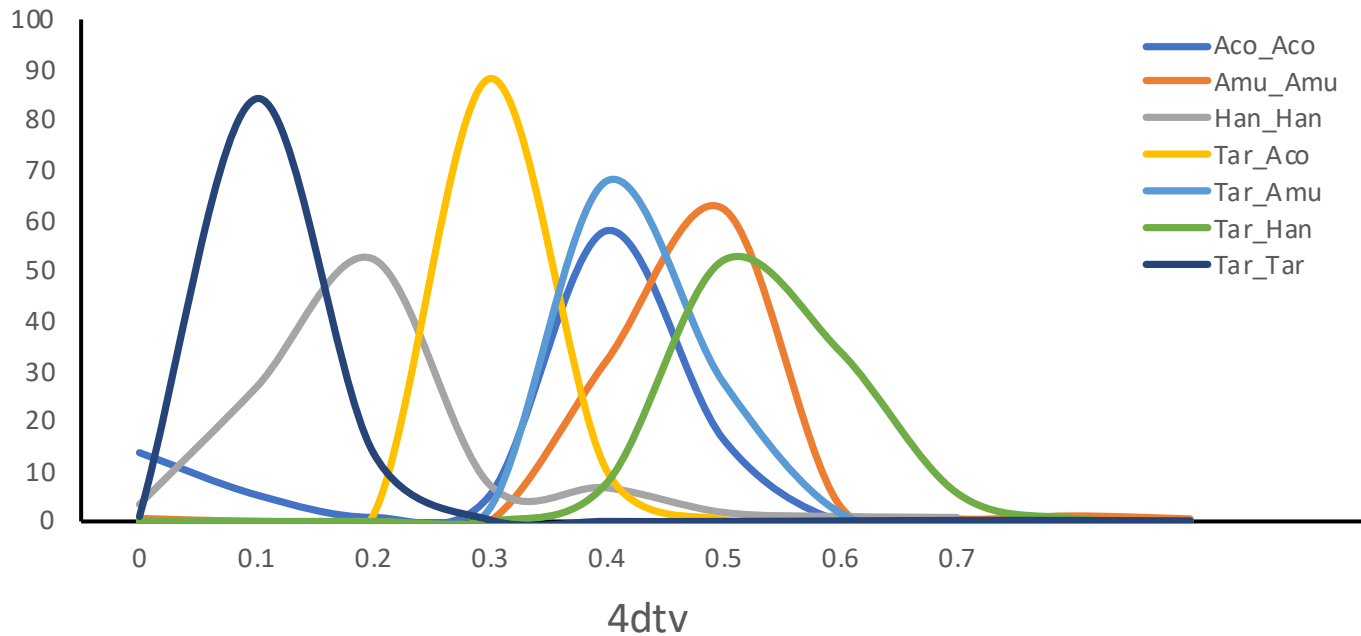

Supplement: giz136_Supplemental_Figures_and_Tables [file giz136_supplemental_figures_and_tables.zip › Supp_Figure_7-4dTv.pdf]
