# Supplementary material for: Trochodendron aralioides, the first chromosome-level draft genome in Trochodendrales and a valuable resource for basal eudicot research
Source: Gigascience. 2019 Nov 18;8(11):giz136. doi: 10.1093/gigascience/giz136 (PMC6859433; doi:10.1093/gigascience/giz136)
Supplement: giz136_Response_to_Reviewer_Comments_Original_Submission [file giz136_response_to_reviewer_comments_original_submission.pdf]

Author's Response To Reviewer Comments

Close

Joeri Strijk, Damien Hinsinger, Feng-Ping Zhang, KunFang Cao 21th August 2019
Biodiversity Genomics Team
Plant Ecophysiology & Evolution Group
Guangxi Key Laboratory of Forest Ecology and Conservation
College of Forestry, Guangxi University, Nanning, Guangxi 530005 PR China

Dear Editor,

Please find attached our revised and improved manuscript entitled "Trochodendron aralioides, the first chromosome-level draft genome in Trochodendrales and a valuable resource for basal eudicot research" by Joeri S. Strijk, Damien D. Hinsinger, Feng-Ping Zhang and KunFang Cao. We have carefully studied the reviewer's recommendations and provide here a detailed point-by-point treatment. Please find our responses to the specific comments below, in italics, and the tracked changes file attached to this letter. The corrected manuscript file has been uploaded separately.

Following suggestions of the reviewers we added technical information and provided explanations for our methodological choices, that followed the state-of-the-art of genome assembly and analysis. We have thoroughly reviewed the text and made improvements to the grammar and spelling of our manuscript. Finally, we addressed specific comments as shown below.

Reviewer reports:
Reviewer #1: The authors provide a high confident genome assembly of Trochodendron aralioides, which is a basal eudicot species next to Amborella and Winteracease.

Reply : Trochodendron is actually a basal eudicot, only distantly related to Amborella and Winteraceae, but much closer from other eudicots and families such as Buxaceae, Ranunculaceae, Berberidaceae, Sabiaceae and Proteaceae. This reviewer's mistake, however, doesn't preclude the validity of our results and/or conclusions.

By providing the first high quality chromosome-level genome assembly of its kind, this study shall contribute greatly to the genome evolution research of eudicot plants. The assemble, annotation, and phylogentic/selection analysese are well performed with clear description. Therefore, I would suggest for publication in gigaScience.

Minor Issues:
1. For functional annotation, the evalue cutoff of 1E-5 seems too low for protein similarity search (BLASTP, Pfam, KEGG etc).

Reply : This value is very standard, as illustrated for the chinese chestnut genome (bioRxiv 615047; doi: https://doi.org/10.1101/615047), as well as in (for example) several recently published studies :
Moreno-Santillán, D. D., Machain-Williams, C., Hernández-Montes, G., & Ortega, J. (2019). De Novo transcriptome Assembly and Functional Annotation in Five species of Bats. Scientific reports, 9(1), 6222.
Leandro Costa Nascimento, Karina Yanagui, Juliana Jose, Eduardo L O Camargo, Maria Carolina B Grassi, Camila P Cunha, José Antonio Bressiani, Guilherme M A Carvalho, Carlos Roberto Carvalho, Paula F Prado, Piotr Mieczkowski, Gonçalo A G Pereira, Marcelo F Carazzolle, Unraveling the complex genome of Saccharum spontaneum using Polyploid Gene Assembler, DNA Research, Volume 26, Issue 3, June 2019, Pages 205–216, https://doi.org/10.1093/dnares/dsz001
Jing Yang, Hafiz Muhammad Wariss, Lidan Tao, Rengang Zhang, Quanzheng Yun, Peter Hollingsworth, Zhiling Dao, Guifen Luo, Huijun Guo, Yongpeng Ma, Weibang Sun, De novo genome assembly of the endangered Acer yangbiense, a plant species with extremely small populations endemic to Yunnan Province, China, GigaScience, Volume 8, Issue 7, July 2019, giz085, https://doi.org/10.1093/gigascience/giz085

Gaorui Gong, Cheng Dan, Shijun Xiao, Wenjie Guo, Peipei Huang, Yang Xiong, Junjie Wu, Yan He, Jicheng Zhang, Xiaohui Li, Nansheng Chen, Jian-Fang Gui, Jie Mei, Chromosomal-level assembly of yellow catfish genome using third-generation DNA sequencing and Hi-C analysis, GigaScience, Volume 7, Issue 11, November 2018, giy120, https://doi.org/10.1093/gigascience/giy120
Therefore, we don't think changing this cut-off value would either improve the manuscript or the reproducibility of the analyses herein.

2. For the ortholog search I think all-against-all OrthoMCL may not perform well with diverged over hundreds millions years. The authors only specified that the longest transcript per locus was selected. I think it would be good to provide more details of the selected orthologs (the number of orthologs selected by OrthoMCL, the distribution of ortholog similarity, how many were used for ML tree inference, how many were used for positive selection analyses PAML, etc).

Reply : OrthoMCL represents the state-of-the-art for ortholog identification, and was used in all above-mentioned papers to identify orthologs (except in the Chestnut manuscript from bioRxiv, but without mentioning any other approach). We added the requested information : number of orthologs (multi-gene families and 1:1 orthologs) and the distribution of their similarity (Supplementary Figure S6a), the number used for phylogenomic inference, positive selection analyses and for dating using PAML.

3. "we used Gblocks [48] to eliminate poorly aligned positions and divergent regions from the alignment ". Please specify what criteria were used for alignment quality control and divergent filtering. Do removing of the most divergent regions change the estimates? Please provide a distribution of Ka/Ks for the genome or 238 genes. I don't think the KEGG results for those 238 genes are significantly enriched for cell metabolism as the adjust p-values are quite high (0.28 or higher, Table S11).

Reply : As the most diverging genes can blur the positive selection signal, and result in false positives (Jordan & Goldman, 2012 in MBE, 29(4): 1125-1139), it is strongly advised to filter them out prior to the analysis. We don't think including error-prone data in a Positive Selection analysis would improve our results and conclusions. We added the parameters used for Gblocks, and improved the phrasing of the 'Positive Selection' part. However, the enrichment was calculated for the genes themselves, for which we got KEGG pathways. The enrichment of the KEGG pathways was not calculated per se. We clarified these sentences as well as the legend of the Table S11, and added the distribution of Ka/Ks values for Trochodendron aralioides (Supplementary Figure 6b).

4. what is the synonymous mutation rate and average Ka/Ks for the species? How these compared to other species, especially the ones in the basal position of eudicot?

Reply : We added the average Ka/KS for Trochodendron aralioides, however such data is apparently not available for the other genomes.

5. Table 2 the last header should be "Combined TEs". It seems a big discrepancy between results of RepeatMasker (TE protein) to those of other two methods.

Reply : We corrected this typo. As highlighted, the last column is not a third method but the combination of the "de novo RepeatModeler, RepeatScout and LTR_FINDER" and "RepeatMasker" approaches. Indeed, adding de novo identified TEs greatly improved the global estimation.

Reviewer #2: This is an informative Data Note MS. The authors successfully assembled Trochodendron aralioides chromosome-scale genome applying multiple high throughput sequencing technologies and platforms. The authors also predicted proteins, estimated divergence time, and investigated the genome-wide duplication events. Most of the MS was well-written. The MS might be improved if the authors would provide minor revisions by responding to the following minor comments.

Minor comments:

1. The MS was provided no page numbers and no line numbers. This results in communication difficulties between the authors, reviewers, and editors.

Reply : We added lines numbers.

2. The script "duplication_rm.v2" is not available. Please provide the script as a supplementary or in a

web link.

Reply : We thanks the reviewer for identifying this oversight on our part. We made it available in the folder “scripts” of the provided data for review (that should be included in GigaDB after acceptance, and thus be publicly available).

3. Several references are missing for data analysis tools, for example: Myer's algorithm, Quiver, and BLAST. It would be better if the authors would provide the references.

Reply : we added the missing references the reviewer highlighted and carefully checked the manuscript for additional missing references.

4. There is some awkward punctuation in the section "Genomic DNA extraction, Illumina sequencing and genome size estimation." Please fix them.

Reply : We fixed the phrasing of this section and carefully checked other sections as well.

5. There are sentence fragments in the section "Annotation." Please fix them.

Reply : We fixed the sentence fragments and improved the general phrasing.

Thank you in advance for considering our resubmitted manuscript. Please do not hesitate to contact us should you require any additional information. We look forward to hearing from you soon.

Yours sincerely,

The authors

Close
