# Supplementary material for: Trochodendron aralioides, the first chromosome-level draft genome in Trochodendrales and a valuable resource for basal eudicot research
Source: Gigascience. 2019 Nov 18;8(11):giz136. doi: 10.1093/gigascience/giz136 (PMC6859433; doi:10.1093/gigascience/giz136)
Supplement: giz136_Response_to_Reviewer_Comments_Revision_1 [file giz136_response_to_reviewer_comments_revision_1.pdf]

Author's Response To Reviewer Comments

Close

Joeri Strijk, Damien Hinsinger, Feng-Ping Zhang, KunFang Cao 18th September 2019
Biodiversity Genomics Team
Plant Ecophysiology & Evolution Group
Guangxi Key Laboratory of Forest Ecology and Conservation
College of Forestry, Guangxi University, Nanning, Guangxi 530005 PR China

Dear Editor,

Please find attached our reply to the review of our manuscript entitled “Trochodendron aralioides, the first chromosome-level draft genome in Trochodendrales and a valuable resource for basal eudicot research” by Joeri S. Strijk, Damien D. Hinsinger, Feng-Ping Zhang and KunFang Cao. We have carefully considered the reviewer’s opinion and provide here a detailed point-by-point treatment of the remaining points. Please find our responses to the specific comments below, in italics.

The reviewer has maintained some of the previously raised remarks, and we have discussed these (together with our sequence facility personal). We have also looked into common-use practices on both of these, to aid in further clarification. Below we provide additional explanation.

Reviewer reports:
Reviewer #1: The authors generally answered my questions together with changes in the text so I think this paper should be accepted for publication. However, I still have to clarify some issues:

1. An E-value cutoff = 1e-5 for protein search means a piece (HSP) of shorter than 20 bp with low identity would pass. And I don't believe this did not change their results. There is no standard for the e-value cutoff as the authors argued and even if there were, I believe it would not be 1e-5. I can make a much longer list of literatures using more stringent and serious cutoffs from different statistics. I am sorry to say that the so called "standard" used in the studies provided by authors is not so serious.

As an example for a randomly picked sequence blastp against NCBI:
Range 1: 145 to 168GenPeptGraphicsNext MatchPrevious Match
Alignment statistics for match #1
Score Expect Identities Positives Gaps
51.1 bits(113) 1e-05 16/24(67%) 17/24(70%) 0/24(0%)
Query 1 CGNETMKILLGAVEVLWAQQEQEW 24
CGNETM IL GA E LW +EQ W
Sbjct 145 CGNETMIILAGALEALWSAHEQNW 168

Reply : We disagree with the reviewer for several reasons :
Homology assessment is only one of the methods we used for gene annotations. As detailed in the manuscript, we also used ab initio and transcriptomes, and the results of these 3 approaches were combined and filtered. Thus any misidentified homology can be expected to be corrected during the combination and filtering steps.
The example the reviewer gave cannot be compared with our results, as we blasted nucleotide sequences, not a protein sequence as exemplified, and that even the corresponding nt sequence would have been dropped out during the filtering step “we filtered out low quality gene models, defined as follows: (1) coding region lengths ≤150 bp” (20 amino-acids=90 nucleotides).
We agree that some other studies have in fact used a more stringent cutoff value, especially when using closely related species. However, when blasting against distant taxa, as in our manuscript (and other genomes-based studies where no closely related species is available), using a cutoff value of 1E-5 is more common, to take into account the bigger expected genetic distances. As already outlined previously, many studies (several of which published in GigaScience or other high impact journals) relied on this cutoff value.

2. I am not sure if adding "de novo identified TEs" improved the estimation or just greatly overestimated the TE% due to e.g. very loose cutoff choice by the authors. It almost triple the size. Simply increase the TE family size cannot be called as "improved"

Reply : The bigger number of de novo identified TEs is due to the different approaches used, as highlighted in both the manuscript and the table legend. To summarize, the TE-proteins were identified using one pipeline that identified ONLY the transposable elements that contain the searched protein domain. On the opposite, the de novo approach used 3 different softwares, based on different methods and algorithms to identify also the non protein TEs. In addition, our values are in the same range than other publications in plants (when different methods are distinguished, e.g. in Ceiba bombax - Gao et al. GigaScience, 7(5), 2018, https://doi.org/10.1093/gigascience/giy051).

Thank you in advance for considering our manuscript. Please do not hesitate to contact us should you require any additional information. We look forward to hearing from you soon.

Yours sincerely,

The authors

Close
