# Supplementary material for: Trochodendron aralioides, the first chromosome-level draft genome in Trochodendrales and a valuable resource for basal eudicot research
Source: Gigascience. 2019 Nov 18;8(11):giz136. doi: 10.1093/gigascience/giz136 (PMC6859433; doi:10.1093/gigascience/giz136)
Supplement: giz136_Reviewer_1_Report_Original_Submission — Jun Chen -- 8/6/2019 Reviewed [file giz136_reviewer_1_report_original_submission.pdf]

Reviewer Report

Title: Trochodendron aralioides, the first chromosome-level draft genome in Trochodendrales and a valuable resource for basal eudicot research

Version: Original Submission **Date:** 8/6/2019

Reviewer name: Jun Chen

Reviewer Comments to Author:

The authors provide a high confident genome assembly of Trochodendron aralioides, which is a basal eudicot species next to Amborella and Winteracease. By providing the first high quality chromosome-level genome assembly of its kind, this study shall contribute greatly to the genome evolution research of eudicot plants. The assemble, annotation, and phylogentic/selection analysese are well performed with clear description. Therefore, I would suggest for publication in gigaScience.
Minor Issues:
1. For functional annotation, the evalue cutoff of 1E-5 seems too low for protein similarity search (BLASTP, Pfam, KEGG etc).
2. For the ortholog search I think all-against-all OrthoMCL may not perform well with diverged over hundreds millions years. The authors only specified that the longest transcript per locus was selected. I think it would be good to provide more details of the selected orthologs (the number of orthologs selected by OrthoMCL, the distribution of ortholog similarity, how many were used for ML tree inference, how many were used for positive selection analyses PAML, etc).
3. "we used Gblocks [48] to eliminate poorly aligned positions and divergent regions from the alignment ". Please specify what criteria were used for alignment quality control and divergent filtering. Do removing of the most divergent regions change the estimates? Please provide a distribution of Ka/Ks for the genome or 238 genes. I don't think the KEGG results for those 238 genes are significantly enriched for cell metabolism as the adjust p-values are quite high (0.28 or higher, Table S11).
4. what is the synonymous mutation rate and average Ka/Ks for the species? How these compared to other species, especially the ones in the basal position of eudicot?
5. Table 2 the last header should be "Combined TEs". It seems a big discrepancy between results of RepeatMasker (TE protein) to those of other two methods.

Level of Interest

Please indicate how interesting you found the manuscript: Choose an item.

Quality of Written English

Please indicate the quality of language in the manuscript: Choose an item.

Declaration of Competing Interests

Please complete a declaration of competing interests, considering the following questions:

- Have you in the past five years received reimbursements, fees, funding, or salary from an organisation that may in any way gain or lose financially from the publication of this manuscript, either now or in the future?
- Do you hold any stocks or shares in an organisation that may in any way gain or lose financially from the publication of this manuscript, either now or in the future?
- Do you hold or are you currently applying for any patents relating to the content of the manuscript?
- Have you received reimbursements, fees, funding, or salary from an organization that holds or has applied for patents relating to the content of the manuscript?
- Do you have any other financial competing interests?
- Do you have any non-financial competing interests in relation to this paper?

If you can answer no to all of the above, write 'I declare that I have no competing interests' below. If your reply is yes to any, please give details below.

I declare that I have no competing interests

I agree to the open peer review policy of the journal. I understand that my name will be included on my report to the authors and, if the manuscript is accepted for publication, my named report including any attachments I upload will be posted on the website along with the authors' responses. I agree for my report to be made available under an Open Access Creative Commons CC-BY license (http://creativecommons.org/licenses/by/4.0/). I understand that any comments which I do not wish to be included in my named report can be included as confidential comments to the editors, which will not be published.

Choose an item.

To further support our reviewers, we have joined with Publons, where you can gain additional credit to further highlight your hard work (see: https://publons.com/journal/530/gigascience). On publication of this paper, your review will be automatically added to Publons, you can then choose whether or not to claim your Publons credit. I understand this statement.

Yes Choose an item.
