# Supplementary material for: Trochodendron aralioides, the first chromosome-level draft genome in Trochodendrales and a valuable resource for basal eudicot research
Source: Gigascience. 2019 Nov 18;8(11):giz136. doi: 10.1093/gigascience/giz136 (PMC6859433; doi:10.1093/gigascience/giz136)
Supplement: giz136_Reviewer_1_Report_Revision_1 — Jun Chen -- 9/6/2019 Reviewed [file giz136_reviewer_1_report_revision_1.pdf]

Reviewer Report

Title: Trochodendron aralioides, the first chromosome-level draft genome in Trochodendrales and a valuable resource for basal eudicot research

Version: Revision 1 **Date:** 9/6/2019

Reviewer name: Jun Chen

Reviewer Comments to Author:

The authors generally answered my questions together with changes in the text so I think this paper should be accepted for publication. However, I still have to clarify some issues:
1. An E-value cutoff = 1e-5 for protein search means a piece (HSP) of shorter than 20 bp with low identity would pass. And I don't believe this did not change their results. There is no standard for the e-value cutoff as the authors argued and even if there were, I believe it would not be 1e-5. I can make a much longer list of literatures using more stringent and serious cutoffs from different statistics. I am sorry to say that the so called "standard" used in the studies provided by authors is not so serious.
As an example for an randomly picked sequence blastp against NCBI:
Range 1: 145 to 168GenPeptGraphicsNext MatchPrevious Match
Alignment statistics for match #1
Score Expect Identities Positives Gaps
51.1 bits(113) 1e-05 16/24(67%) 17/24(70%) 0/24(0%)
Query 1 CGNETMKILLGAVEVLWAQQEQEW 24
CGNETM IL GA E LW +EQ W
Sbjct 145 CGNETMIILAGALEALWSAHEQNW 168
2. I am not sure if adding "de novo identified TEs" improved the estimation or just greatly overestimated the TE% due to e.g. very loose cutoff choice by the authors. It almost triple the size. Simply increase the TE family size cannot be called as "improved"

Level of Interest

Please indicate how interesting you found the manuscript: Choose an item.

Quality of Written English

Please indicate the quality of language in the manuscript: Choose an item.

Declaration of Competing Interests

Please complete a declaration of competing interests, considering the following questions:

- Have you in the past five years received reimbursements, fees, funding, or salary from an organisation that may in any way gain or lose financially from the publication of this manuscript, either now or in the future?
- Do you hold any stocks or shares in an organisation that may in any way gain or lose financially from the publication of this manuscript, either now or in the future?
- Do you hold or are you currently applying for any patents relating to the content of the manuscript?
- Have you received reimbursements, fees, funding, or salary from an organization that holds or has applied for patents relating to the content of the manuscript?
- Do you have any other financial competing interests?
- Do you have any non-financial competing interests in relation to this paper?

If you can answer no to all of the above, write 'I declare that I have no competing interests' below. If your reply is yes to any, please give details below.

I declare that I have no competing interests

I agree to the open peer review policy of the journal. I understand that my name will be included on my report to the authors and, if the manuscript is accepted for publication, my named report including any attachments I upload will be posted on the website along with the authors' responses. I agree for my report to be made available under an Open Access Creative Commons CC-BY license (http://creativecommons.org/licenses/by/4.0/). I understand that any comments which I do not wish to be included in my named report can be included as confidential comments to the editors, which will not be published.

Choose an item.

To further support our reviewers, we have joined with Publons, where you can gain additional credit to further highlight your hard work (see: https://publons.com/journal/530/gigascience). On publication of this paper, your review will be automatically added to Publons, you can then choose whether or not to claim your Publons credit. I understand this statement.

Yes Choose an item.
