# Supplementary material for: Trochodendron aralioides, the first chromosome-level draft genome in Trochodendrales and a valuable resource for basal eudicot research
Source: Gigascience. 2019 Nov 18;8(11):giz136. doi: 10.1093/gigascience/giz136 (PMC6859433; doi:10.1093/gigascience/giz136)
Supplement: giz136_Reviewer_2_Report_Original_Submission — Shih-Hui Liu -- 8/18/2019 Reviewed [file giz136_reviewer_2_report_original_submission.pdf]

Reviewer Report

Title: Trochodendron aralioides, the first chromosome-level draft genome in Trochodendrales and a valuable resource for basal eudicot research

Version: Original Submission **Date:** 8/18/2019

Reviewer name: Shih-Hui Liu

Reviewer Comments to Author:

This is an informative Data Note MS. The authors successfully assembled Trochodendron aralioides chromosome-scale genome applying multiple high throughput sequencing technologies and platforms. The authors also predicted proteins, estimated divergence time, and investigated the genome-wide duplication events. Most of the MS was well-written. The MS might be improved if the authors would provide minor revisions by responding to the following minor comments.
Minor comments:
1. The MS was provided no page numbers and no line numbers. This results in communication difficulties between the authors, reviewers, and editors.
2. The script "duplication_rm.v2" is not available. Please provide the script as a supplementary or in a web link.
3. Several references are missing for data analysis tools, for example: Myer's algorithm, Quiver, and BLAST. It would be better if the authors would provide the references.
4. There is some awkward punctuation in the section "Genomic DNA extraction, Illumina sequencing and genome size estimation." Please fix them.
5. There are sentence fragments in the section "Annotation." Please fix them.

Level of Interest

Please indicate how interesting you found the manuscript: Choose an item.

Quality of Written English

Please indicate the quality of language in the manuscript: Choose an item.

Declaration of Competing Interests

Please complete a declaration of competing interests, considering the following questions:

- Have you in the past five years received reimbursements, fees, funding, or salary from an organisation that may in any way gain or lose financially from the publication of this manuscript, either now or in the future?

- Do you hold any stocks or shares in an organisation that may in any way gain or lose financially from the publication of this manuscript, either now or in the future?
- Do you hold or are you currently applying for any patents relating to the content of the manuscript?
- Have you received reimbursements, fees, funding, or salary from an organization that holds or has applied for patents relating to the content of the manuscript?
- Do you have any other financial competing interests?
- Do you have any non-financial competing interests in relation to this paper?

If you can answer no to all of the above, write 'I declare that I have no competing interests' below. If your reply is yes to any, please give details below.

I declare that I have no competing interests

I agree to the open peer review policy of the journal. I understand that my name will be included on my report to the authors and, if the manuscript is accepted for publication, my named report including any attachments I upload will be posted on the website along with the authors' responses. I agree for my report to be made available under an Open Access Creative Commons CC-BY license (http://creativecommons.org/licenses/by/4.0/). I understand that any comments which I do not wish to be included in my named report can be included as confidential comments to the editors, which will not be published.

Choose an item.

To further support our reviewers, we have joined with Publons, where you can gain additional credit to further highlight your hard work (see: https://publons.com/journal/530/gigascience). On publication of this paper, your review will be automatically added to Publons, you can then choose whether or not to claim your Publons credit. I understand this statement.

Yes Choose an item.
